# Association of Zinc Finger Antiviral Protein Binding to Viral Genomic RNA with Attenuation of Replication of Echovirus 7

**DOI:** 10.1128/mSphere.01138-20

**Published:** 2021-01-06

**Authors:** Niluka Goonawardane, Dung Nguyen, Peter Simmonds

**Affiliations:** a Nuffield Department of Medicine, University of Oxford, Oxford, United Kingdom; University Medical Center Freiburg

**Keywords:** CpG dinucleotide, UpA dinucleotide, attenuation, echovirus 7, oligoadenylate synthetase 3, replicon, zinc antiviral protein

## Abstract

Previous studies have implicated both zinc finger antiviral protein (ZAP) and oligoadenylate synthetase 3 (OAS3)/RNase L in the attenuation of RNA viruses with elevated CpG and UpA dinucleotides. Mechanisms and interrelationships between these two pathways were investigated using an echovirus 7 (E7) replicon with compositionally modified sequences inserted into the 3′ untranslated region. ZAP and OAS3 immunoprecipitation (IP) assays provided complementary data on dinucleotide composition effects on binding. Elevated frequencies of alternative pyrimidine/purine (CpA and UpG) and reversed (GpC and ApU) dinucleotides showed no attenuating effect on replication or specific binding to ZAP by IP. However, the bases 3′ and 5′ of CpG motifs influenced replication and ZAP binding; UCGU enhanced CpG-mediated attenuation and ZAP binding, while A residues shielded CpGs from ZAP recognition. Attenuating effects of elevated frequencies of UpA on replication occurred independently of CpG dinucleotides and bound noncompetitively with CpG-enriched RNA, consistent with a separate recognition site from CpG. Remarkably, immunoprecipitation with OAS3 antibody reproduced the specific binding to CpG- and UpA-enriched RNA sequences. However, OAS3 and ZAP were coimmunoprecipitated in both ZAP and OAS3 IP and colocalized with E7 and stress granules (SGs) by confocal microscopy analysis of infected cells. ZAP’s association with larger cellular complexes may mediate the recruitment of OAS3/RNase L, KHNYN, and other RNA degradation pathways.

**IMPORTANCE** We recently discovered that the OAS3/RNase L antiviral pathway is essential for restriction of CpG- and UpA-enriched viruses, in addition to the requirement for zinc finger antiviral protein (ZAP). The current study provides evidence for the specific dinucleotide and wider recognition contexts associated with virus recognition and attenuation. It further documents the association of ZAP and OAS3 and association with stress granules and a wider protein interactome that may mediate antiviral effects in different cellular compartments. The study provides a striking reconceptualization of the pathways associated with this aspect of antiviral defense.

## Abstract (precis)

We recently discovered that the OAS3/RNase L antiviral pathway is essential for restriction of CpG- and UpA-enriched viruses, in addition to the requirement for zinc finger antiviral protein (ZAP). The current study provides evidence for the specific dinucleotide and wider recognition contexts associated with virus recognition and attenuation.

## INTRODUCTION

It is increasingly recognized that the nucleotide composition of the genomes of viruses has a profound influence on their subsequent interaction with the cell upon infection. Elevated frequencies of the CpG dinucleotide in RNA virus genomes or in mRNAs expressed during replication substantially modify replication kinetics and gene expression in mammalian viruses, including enteroviruses (1) and other RNA viruses (2–5), HIV-1 (6–8), and other retroviruses (9), and hepatitis B virus (10), through their interactions with zinc finger antiviral protein (ZAP). ZAP has been shown to specifically bind to high-CpG RNA sequences (1, 8) and activate a range of RNA degradation pathways or induce translational arrest. Among its many documented effects, ZAP binding may inhibit cap-dependent translation (4, 11) and decap mRNA sequences, making them susceptible to 5′-3′ degradation by the cellular nuclease Xrn1 (12, 13). ZAP additionally interacts with stress granule-associated components (14–16), which may potentially sequester viral mRNAs to repress translation. Recently, it was shown that ZAP binding to CpG-enriched mRNA sequences of HIV-1 activates a cellular nuclease, KHNYN, that degrades viral RNAs (6) and is required for its restriction of HIV-1 replication. In addition to KHNYN, ZAP function is additionally dependent on TRIM25, a member of the tripartite motif family of E3 ubiquitin ligases (8, 17–19).

We recently obtained evidence that the attenuation of high-CpG mutants of the enterovirus echovirus 7 (E7) is further dependent on oligoadenylate synthetase 3 (OAS3) and the downstream activity of RNase L (1, 20). Reversal of attenuation was observed in both RNase L and OAS3 CRISPR (clustered regularly interspaced palindromic repeat) knockout (KO) cell lines despite the abundant expression of ZAP in these cell lines. However, we have not established how or whether high-CpG sequences might be recognized by OAS3 and then degraded by RNase L or the nature of the interaction of ZAP with the antiviral pathway. The targets of OAS3 (and other OASs) are long double-stranded-RNA (dsRNA) duplexes (21) without any substantial dependence on the RNA sequence for binding, making it unlikely that OAS3 directly recognizes and specifically binds to high-CpG RNA sequences. We speculated that OAS3 and RNase L form part of an alternative downstream antiviral pathway activated by ZAP, although the complete reversal of CpG-mediated attenuation of E7 in OAS3 KO cells is seemingly at odds with the demonstrated role of KHNYN downstream of ZAP in HIV-1 restriction (6).

In addition to the growing complexity of the antiviral pathways mediated by ZAP binding, we have additionally shown that RNA sequences with elevated frequencies of the UpA dinucleotide may additionally be targeted for ZAP binding and virus restriction (1). UpA-mediated restriction in E7 was moreover similarly reversed in ZAP, OAS3, and RNase L KO cell lines. RNA transcripts of an E7 viral cDNA with a high-UpA insert in the coding region showed 1,000-fold-greater binding to ZAP than wild-type (WT) sequences, similar to that observed for a similarly constructed high-CpG viral RNA (1). While cocrystallization of ZAP with high-CpG ligands (22, 23) recently provided information on the interaction of the zinc finger domains with CpG dinucleotides (and the stoichiometry of bases surrounding the motif), it is conceivable that UpA, as a similarly shaped pyrimidine (Y)-purine (R) dinucleotide, might fit into the binding site. This degree of target flexibility would imply that other YpR dinucleotides (UpG and CpA) might be also recognized. An alternative possibility is that ZAP possesses an alternative recognition domain or an indirect mechanism of interaction with UpA dinucleotides.

In the current study, we made extensive use of our recently developed E7 replicon construct (20) in which compositionally modified sequences can be inserted into the 3′ untranslated region (UTR). Because inserted sequences are not translated, any attenuation they produce cannot be mediated through previously proposed effects of CpG (and UpA) sequence modification to produce disfavored codon pairs and reduce translation efficiency (24–28). The system additionally provides much greater freedom to manipulate sequence composition of the inserts, since they do not need to retain the amino acid coding of the native sequence. This restriction greatly limits the mutagenesis possible in currently described virus models. The replicon system allowed us to investigate effects of modification of other dinucleotide frequencies, specifically the alternative YpR dinucleotides and reversed dinucleotides (GpC and ApU), and to analyze the effects of altering the upstream and downstream bases (context) of CpG dinucleotides. The latter was motivated by the possibility of a broader recognition motif for ZAP binding. Effects of sequence modifications on replicon replication were compared with binding affinities of the replicon RNAs for ZAP and OAS3 in an *in vitro* binding assay. This assay enabled further studies of the site specificity of CpG and UpA binding to ZAP and the existence of shared or separate binding sites. Finally, through the use of cell lysates from ZAP and OAS3 KO cell lines, we were able to investigate the interdependence of ZAP and OAS3 on RNA binding and their potential cellular interactions.

## RESULTS

### Alternative dinucleotides in virus attenuation.

**FIG 1:**
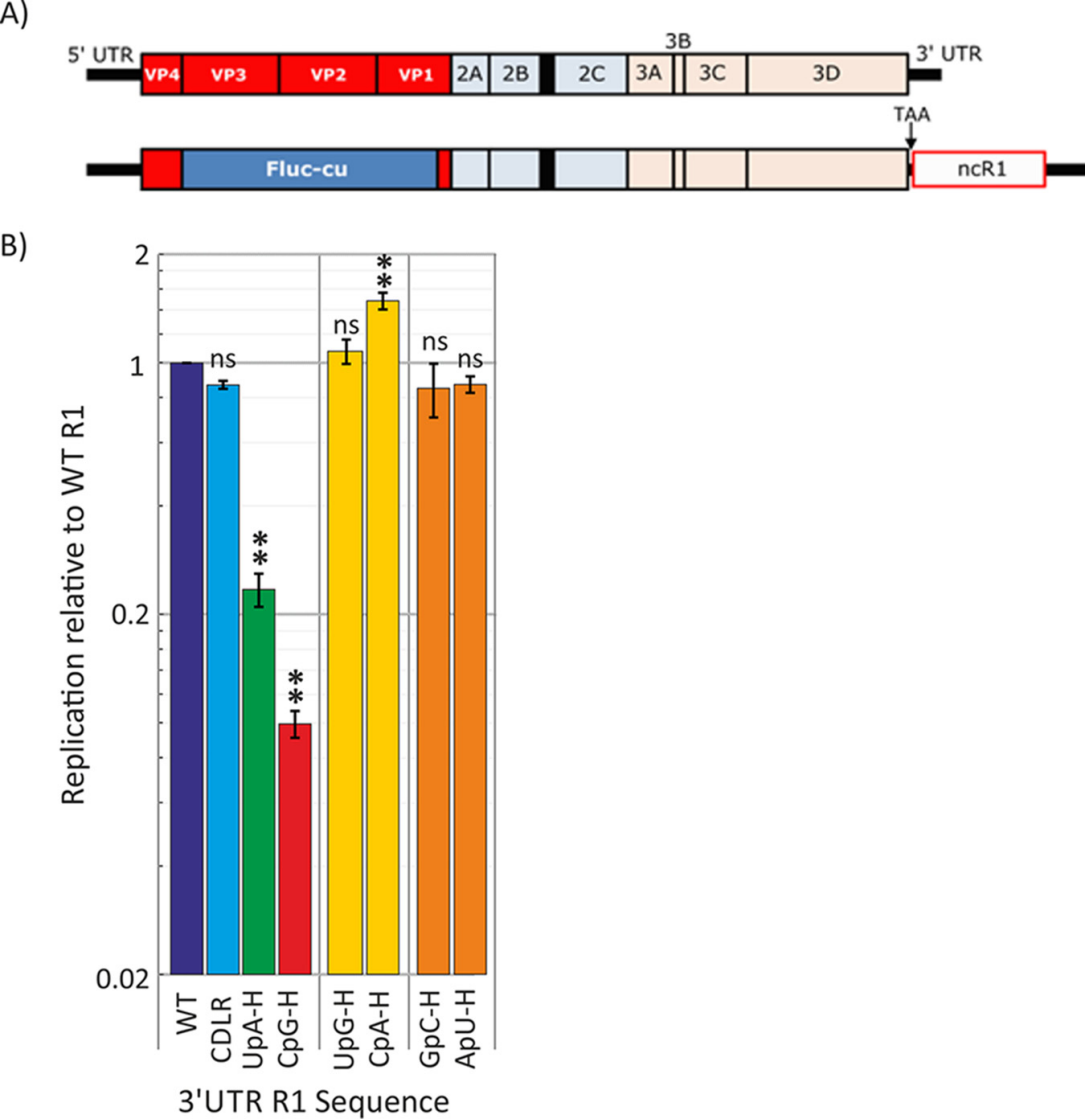
(A) Schematic representation of genome organization of E7 virus (top) and the replicon with noncoding synthetic region R1 (ncR1) inserted into the 3′ UTR. (B) Replication of the WT, permuted control CDLR, and replicons in which the ncR1 region was modified to increase frequencies of CpG, UpA, the other pyrimidine/purine dinucleotides (UpG and CpA), and reversed dinucleotides (GpG and ApU). Replication was measured by luciferase expression at 6 h p.t. and expressed as the ratio of replicon luminescence to that of a replicon with the E7 WT ncR1 sequence (normalized to 1.0). Bar heights represent the means for two biological replicates (each the mean of three technical replicates). Error bars show standard deviations (SDs). The significance of differences from the WT construct was determined by ordinary one-way ANOVA with Bonferroni's correction. **, *P* < 0.0003; ns, no statistically significant difference from WT.

Replication kinetics of E7 replicons are largely unaffected by genome length (20), enabling effects of relatively large insertions of compositionally modified sequence on attenuation to be determined. The current study made extensive use of the replicon construct with a 1,242-nucleotide sequence inserted into the 3′ UTR (ncR1) (Fig. 1A) (20). Insertion of an ncR1 sequence with a native E7 coding sequence or a permuted sequence (referred to as CDLR) with wild-type frequencies of CpG and UpA dinucleotides had little or no effect on replication compared to the unmodified replicon. However, constructs containing sequences with increased CpG or UpA frequencies showed marked attenuation in replication kinetics. Using this model, we investigated whether elevated frequencies of CpA and UpG dinucleotides attenuated replication similarly to the alternative YpR dinucleotides, UpA and CpG. Sequences with maximized frequencies of each were synthesized (Table 1); these contained approximately 3-fold more CpA or UpG dinucleotides than the WT sequence, the maximum possible while keeping mononucleotide frequencies and those of UpA and CpG constant.

**TABLE 1:** Composition of 3′ UTR and coding region mutants of E7

Mutant category and region mutated	Mutant	Coding^*a*^	Length	f_G+C (%)^*b*^	Dinucleotide totals^*c*^					
					CpG	UpA	ApU	GpC	CpA	UpG
Standard composition mutants										
3′ UTR	WT	Yes	1233	47.6	51	62	85	70	130	92
3′ UTR	CDLR	No	1233	47.6	51	62	85	70	130	92
3′ UTR	CpG-H	Yes	1233	56.5	**180**	52	64	116	78	51
3′ UTR	UpA-H	Yes	1233	40.9	39	**171**	114	53	76	69

Reversed dinucleotides										
3′ UTR	ApU-Max	No	1233	47.6	51	62	**205**	70	122	97
3′ UTR	GpC-Max	No	1233	47.6	51	62	85	**180**	118	102

Alternative pyrimidine purine dinucleotides										
3′ UTR	CpA-Max	No	1233	47.6	51	62	114	96	**191**	91
3′ UTR	UpG-Max	No	1233	47.6	51	62	104	91	130	**171**

Altered CpG 5′ and 3′ base contexts										
3′ UTR	ACGA (WT)	No	1233	47.6	51	62	76	47	87	91
3′ UTR	CCGC (WT)	No	1233	47.6	51	62	85	96	89	90
3′ UTR	GCGG (WT)	No	1233	47.6	51	62	83	83	109	64
3′ UTR	UCGU (WT)	No	1233	47.6	51	62	73	47	105	70
3′ UTR	ACGA_CpG-H	No	1233	47.6	**102**	62	80	44	72	88
3′ UTR	CCGC_CpG-H	No	1233	48.1	**102**	62	97	117	52	87
3′ UTR	GCGG_CpG-H	No	1233	46.9	**102**	62	102	119	103	37
3′ UTR	UCGU_CpG-H	No	1233	47.6	**102**	62	66	28	102	46
3′ UTR	XCGX^*d*^	No	1233	54.4	**102**	62	65	89	56	95

Virus coding region mutants										
R1	CpG-H/UpA-L	Yes	1233	56.2	**179**	**19**	62	102	95	53
R1	CpG-L/UpA-H	Yes	1233	41.0	**0**	**170**	114	52	82	84
R1	CpG/UpA-L	Yes	1233	47.5	0	19	81	67	180	125

a Retention of native coding.

b f_G+C, frequency of G and C nucleotides.

c Total of dinucleotides or base frequencies of mutated sequences. Boldface indicates deliberately altered values.

d X indicates any random nucleotide on either side of the CpG dinucleotide.

We additionally investigated whether the attenuation created by high-CpG and high-UpA mutants originates from the resulting increased frequency of self-complementary base pairs in viral genomic RNA or mRNAs. Two additional mutants were generated with sequences containing maximized frequencies of reversed dinucleotides (GpC and ApU). These retained native frequencies of CpG and UpA, but their altered composition would have a similar effect on RNA structure formation in the virus (or replicon) genome. Bioinformatically, increasing the frequencies of GpC and ApU dinucleotides substantially increased the minimum free energy (MFE) on folding predicted by RNAFold from −60.9 kcal/mol of the WT sequences to −72.4 and −78.5 kcal/mol of the high-ApU (ApU-H) and GpC-H mutants, respectively. These were comparable to values of −76.4 and −69.9 kcal/mol of the equivalently mutated UpA-H and CpG-H sequences.

RNA from both sets of mutants was transcribed from linearized replicon cDNA clones along with previously described WT, CpG-H, and UpA-H mutants as controls (20). RNA was transfected into A549 cells, and replication was monitored by luciferase expression at different time points posttransfection (p.t.). At 6 h p.t., there were approximately 20- and 4-fold differences in luciferase expression between the WT replicon and those with the high-CpG and high-UpA inserted sequences (Fig. 1B). At this time point, however, replication of all 4 newly constructed mutants (ApU-Max, GpC-Max, CpA-Max, and UpG-Max) was comparable to that of the WT E7 replicon. The template sequences created to maximize frequencies of the four dinucleotides were used to produce CpG and high-UpA mutants that were otherwise equivalent in sequence composition (Fig. S1). In contrast to replicons with ApU-Max, GpC-Max, CpA-Max and UpG-Max inserts, the replication of both sets of CpG-Max and UpA-Max sequences was attenuated to a degree similar to that of the original, coding-preserved CpG-H and UpA-H mutants (Fig. S1).

10.1128/mSphere.01138-20.3 FIG S1 Replication abilities of CpG context mutants in RD cells. Replication of replicons with 3′ UTRs of different dinucleotide compositions (CpG-H, UpA-H, and a permuted control, CDLR) in RD cells compared to a WT R1 sequence. Replication was monitored by luciferase expression and normalized to WT replication. Bars represent the means for two biological replicates, each with three technical replicates; error bars show standard errors for the biological repeats. ns, no statistically significant difference from the WT 51 CpGs construct; ****, *P* < 0.0001. Download FIG S1, PDF file, 0.2 MB. Copyright © 2021 Goonawardane et al. 2021 Goonawardane et al. This content is distributed under the terms of the Creative Commons Attribution 4.0 International license.

Together, these findings provide no evidence for recognition and downstream potential ZAP-mediated restriction of replication of the CpA and UpG mutants. Furthermore, increased frequencies of GpC and ApU dinucleotides on RNA configuration have no effect on their replication, and this mechanism is unlikely to account for the attenuation of the high-CpG and high-UpA mutants.

### Effects of 5′ and 3′ base contexts on CpG-mediated attenuation.

To investigate whether upstream and downstream contexts influenced CpG-mediated attenuation of the E7 replicon, two sets of modified synthetic sequences with A, C, G, or U bases surrounding each CpG dinucleotide were created. The first set contained the number (*n* = 51) and positions (and therefore spacings) of CpG dinucleotides found in the native R1 sequence; a second set was generated in which the number of CpG dinucleotides was doubled, each in its specific 5′ and 3′ contexts.

**FIG 2:**
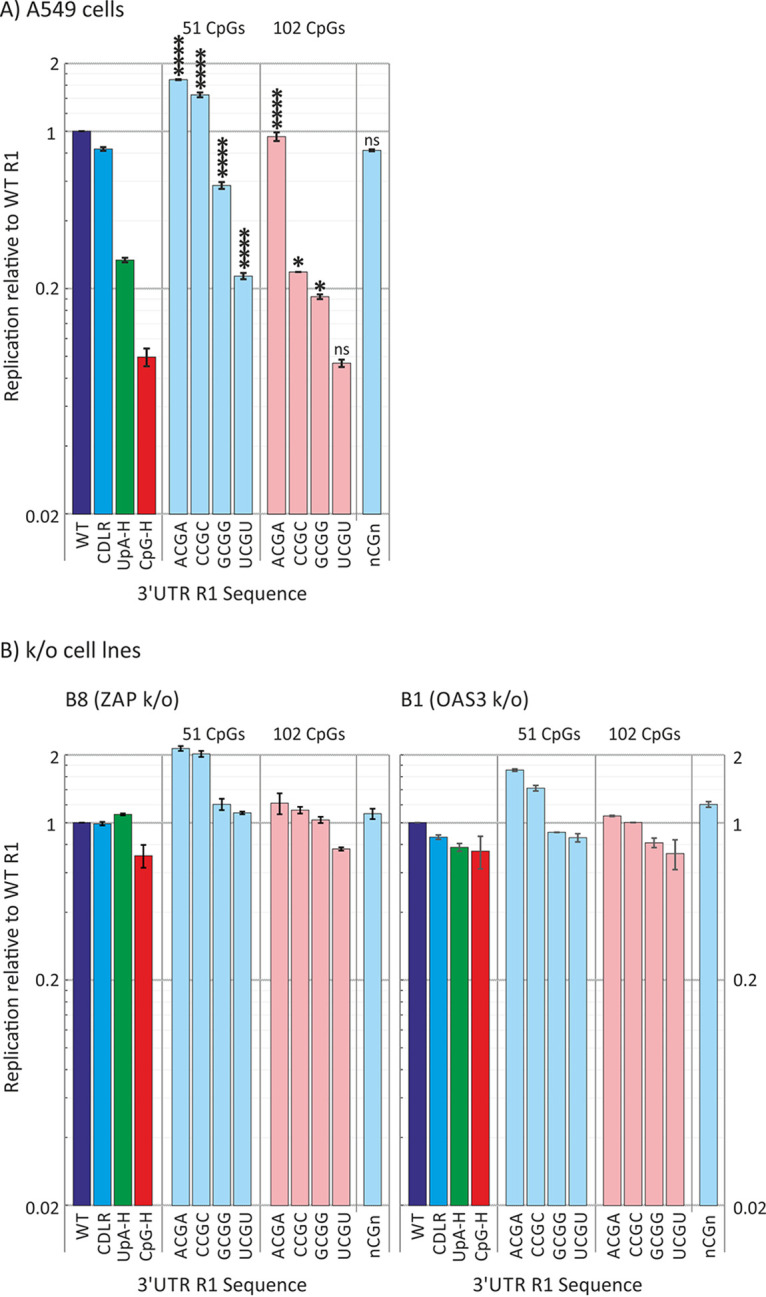
Replication of replicons with R1 sequences containing WT or increased numbers of CpG dinucleotides (*n* = 51 and *n* = 102, respectively) with the specified 5′ and 3′ bases (contexts): A, C, G, T, or n (randomized). Replication at 6 h was normalized to WT replication (WT = 1.0). CpG-H and UpA-h mutants that previously showed attenuated replication were included as controls. Bars represent the means for two biological replicates, each with three technical replicates; error bars show SDs. (A) Replication in A549 cells. The significance of differences from WT was calculated as described for Fig. 1. ****, *P* < 0.0001; *, *P* < 0.04; ns, no statistically significant difference. (B) The equivalent experiment performed in ZAP and OAS3 KO cells.

Transfection of RNA transcripts from these constructs revealed marked differences in replication kinetics, which was strongly influenced by CpG sequence context (Fig. 2A). For both sets of mutants (51 and 102 CpG dinucleotides), those with CpGs surrounded with U residues were substantially more attenuated that those with CpGs in an A context, with attenuation in C and G contexts being intermediate. Remarkably, surrounding CpG with U residues was sufficient to profoundly restrict the replication of the mutant with WT numbers of CpGs (*n* = 51). Conversely, surrounding CpGs with A residues prevented any attenuation even of the high-CpG mutant (*n* = 102). A similar but even more extreme context effect on attenuation was observed in RD cells (Fig. S2)

10.1128/mSphere.01138-20.4 FIG S2 Specificity of amplification of CpG-H and UpA-H R1 RNA sequences. Amplification efficiency of CpG-H and UpA-H R1 transcripts by specific primers used for transcript quantitation in the competition assay (Fig. 8). Amplification of the homologous target was highly efficient, while little or no amplification was observed with mismatched templates (cycle threshold [*C_T_*] values of 35 for amplification of 10^7^ copies of the CpG-H R1 template with UpA-H-specific primers (A) and >40 for amplification of 10^7^ copies of the UpA-H R1 template with CpG-H-specific primers (B). Download FIG S2, PDF file, 0.1 MB. Copyright © 2021 Goonawardane et al. 2021 Goonawardane et al. This content is distributed under the terms of the Creative Commons Attribution 4.0 International license.

To investigate the roles of ZAP and OAS3 in the varied attenuation phenotypes of these mutants, the transfections were repeated in B8 (ZAP KO) and B1 (OAS3 KO) cells (Fig. 2B). For both cell lines, replication differences between mutants were eliminated, providing an indication that the contexts of the CpG dinucleotides influenced their recognition by ZAP (and OAS3) and that the differences in replication did not arise through an alternative restriction mechanism.

**FIG 3:**
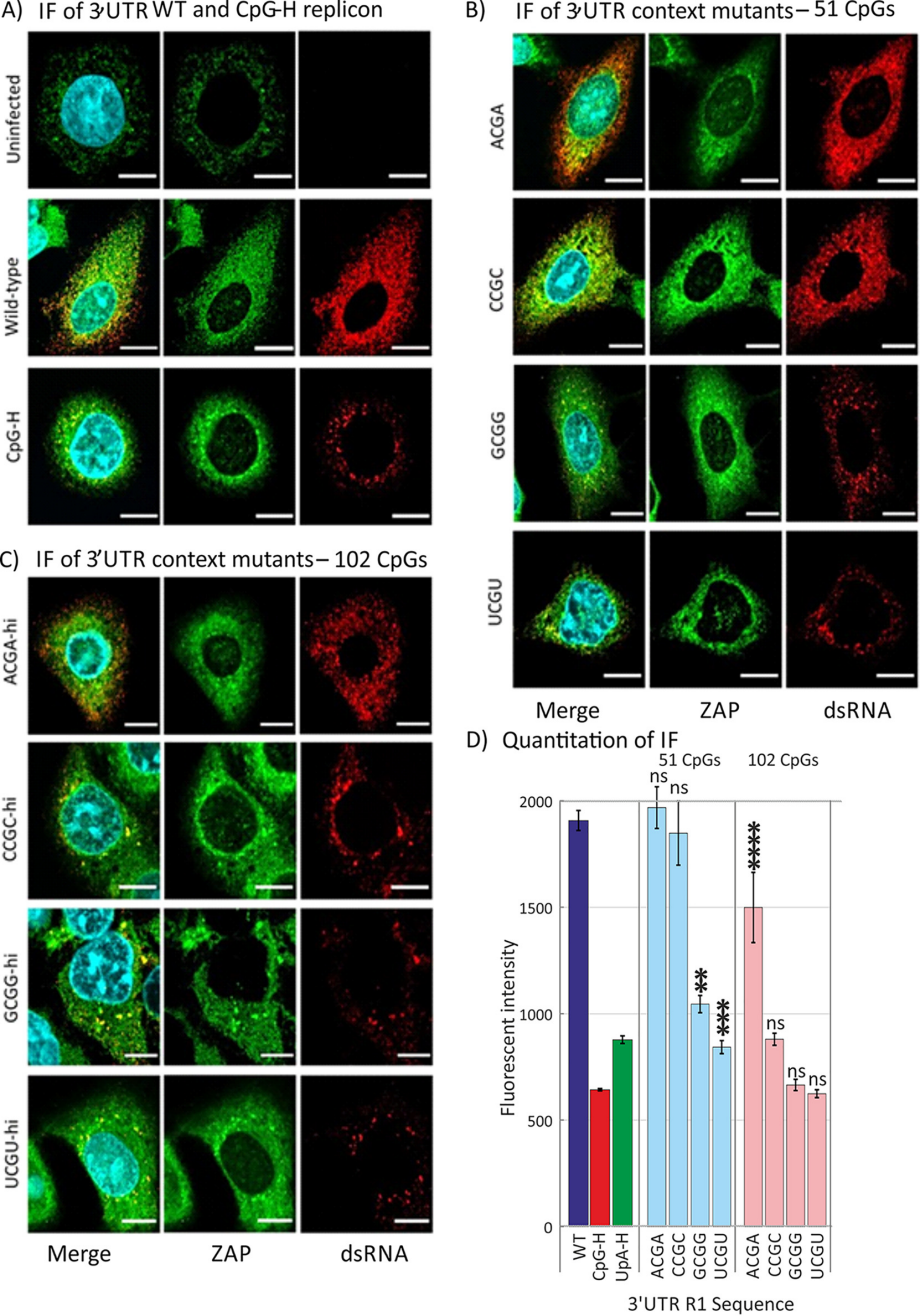
(A) Uninfected A549 cells costained for ZAP by specific antibody and Alexa Fluor 488-coupled secondary antibody and for E7 RNA by J2 (assay specificity control; not detected). WT- or CpG-H (assay positive control)-transfected cells costained for ZAP (green) and for dsRNA detected (red) by J2 antibodies in cells at 4 h posttransfection. Nuclear DNA was stained by DAPI (blue). (B and C) 3′ UTR mutant sequences with either (B) WT level (*n* = 51) or (C) elevated (*n* = 102) CpG frequencies. Bars, 10 μm. (D) Quantitation of fluorescence intensity of infected cells by Airyscan post-acquisition analysis in representative fields of cell monolayers transfected with E7 WT and compositionally modified mutants of E7. Bar heights show the means for two biological replicates; error bars show SDs. Significances of differences from WT are indicated as follows: **, *P* = 0.0015; ***, *P* = 0.0002; ****, *P* < 0.0001; ns, not significant (*P* > 0.05).

The effects of sequence context on CpG-mediated attenuation were similarly apparent when replication was assayed by immunofluorescent detection of dsRNA in infected cells (Fig. 3). The replicon with a WT sequence inserted into the 3′ UTR showed high levels of accumulation of dsRNA and induction of ZAP expression at 4 h p.t. Transfection of A549 cells with the CpG-H and UpA-H mutants induced far less dsRNA, consistent with their attenuation of replication in other assays (Fig. 1B). The different degrees of attenuation of the context mutants (ACGA, CCGC, GCGG, and UCGU) in the replication assay (Fig. 2) were reflected in amounts of dsRNA detected by immunofluorescence (IF) (Fig. 3B and C). Quantitation of dsRNA staining intensity by Airyscan ranked dsRNA detection comparably to replication (Fig. 3D).

### Influence of dinucleotide composition on RNA binding to ZAP.

**FIG 4:**
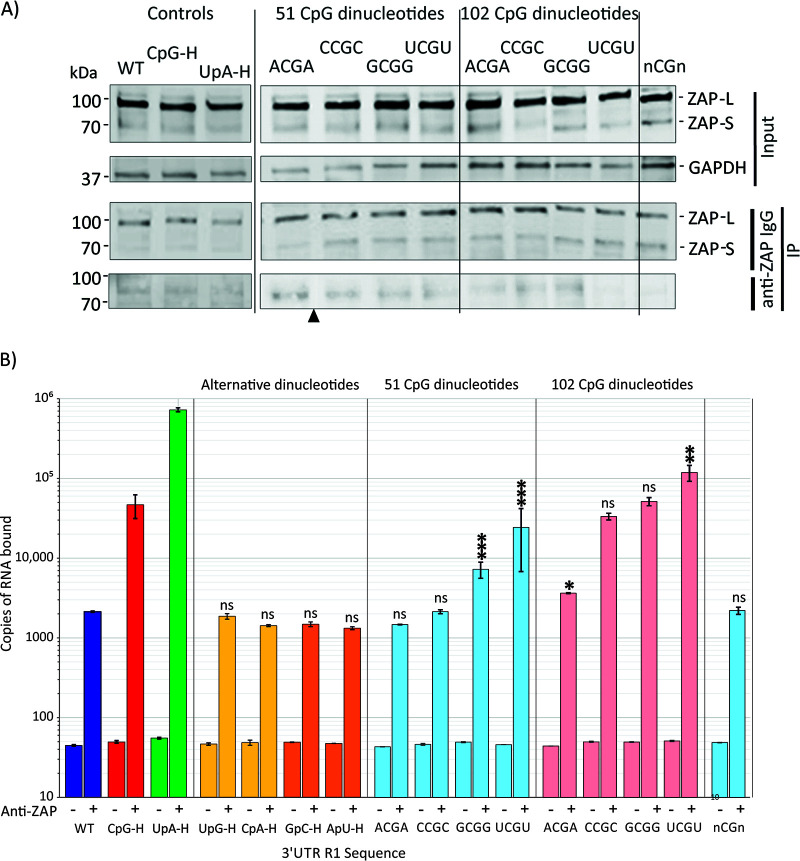
Binding of ZAP to WT, UpA-H, and CpG-H controls or 3′ UTR mutant RNA transcripts. (A) WB of input long (L) and short (S) isoforms of ZAP (~100 kDa and ~70 kDa, respectively) and GAPDH in uninfected cell lysates detected by immunostaining with anti-ZAP polyclonal antibody or GAPDH polyclonal antibody. Cell lysates from replicon-transfected cells were incubated with anti-ZAP antibody or control antibody (IgG) and immobilized to columns with anti-rabbit IgG. The nitrocellulose membrane was damaged at positions indicated by the black arrowhead. The lower panel shows a WB for immunoprecipitated ZAP. (B) Quantitation of E7 viral RNA transcripts with WT, CpG-H, and UpA-H or 3′ UTR mutant sequences by qPCR in immunoprecipitated ZAP (right column in each pair) or mock-precipitated control (left column). RNA was quantified by qPCR using conserved primers and probe from the 5′ UTR (Table S2). Bar heights and error bars represent means and SDs for two biological replicates. The significance of differences from the WT construct is indicated as follows: ***, *P* = 0.0002; **, *P* = 0.0015; *, *P* < 0.015; ns, not significant (*P* > 0.05).

We applied a previously described *in vivo* binding assay for ZAP (1) in which viral RNA sequences transfected into cells were extracted into a cell lysate and the fraction bound to ZAP was estimated through immune capture with an anti-ZAP antibody (Fig. 4). The CpG-H and UpA-H controls showed enhanced binding to ZAP compared to the WT replicon, consistent with previous observations (1). As observed previously, the UpA-H sequence bound more avidly than CpG-H. Replicon mutants with the alternative YpR (CpA and UpG) or reversed (GpC and ApU) dinucleotides showed binding similar to that of the WT replicon, consistent with their lack of attenuation of replication in A549 cells (Fig. 2A). However, the binding or replicon RNA with the CpG dinucleotides placed in different contexts showed markedly different binding, greatest for the UCGU motif and least with ACGA. The findings recapitulate the previous observations of their relative attenuation, including the finding that the mutant with elevated frequencies of CpG motifs surrounded by A residues bound similarly to the WT control, while those with the UCGU motif bind >10-fold more strongly to ZAP even with the WT number of CpG dinucleotides. From these observations, it seems that effects of sequence context on attenuation may primarily originate from effects on ZAP binding.

**FIG 5:**
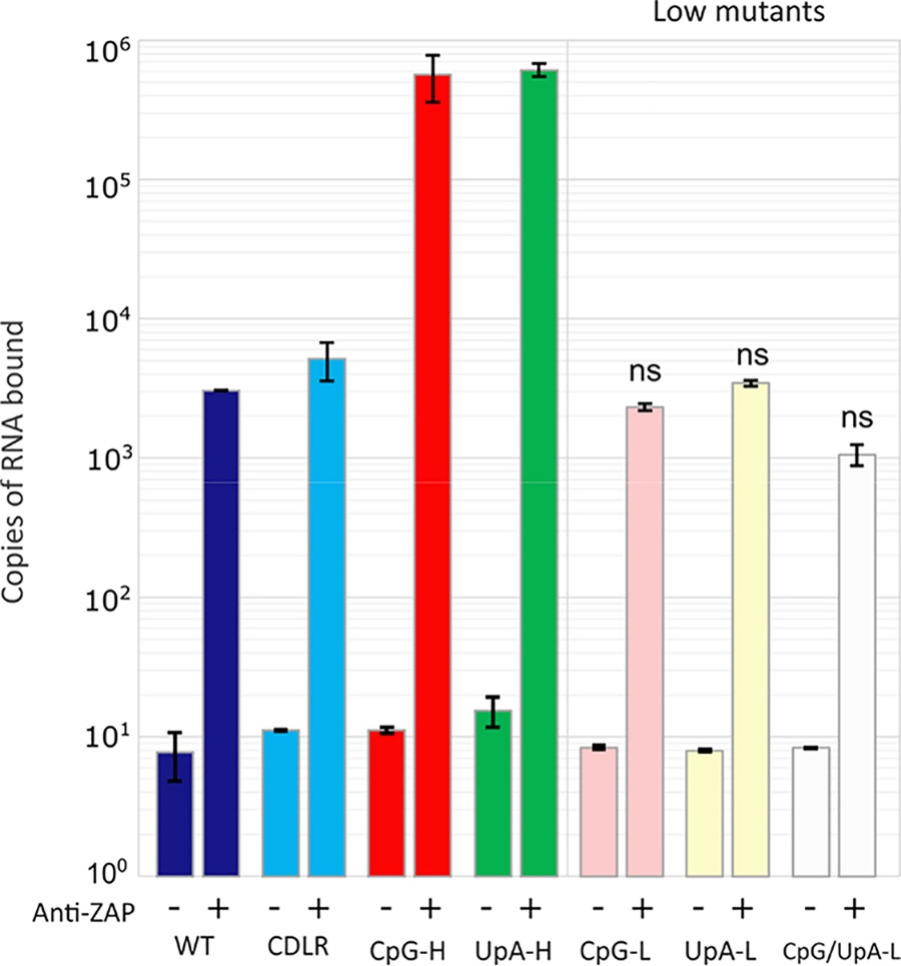
Binding of ZAP in IFN-β-prestimulated cell lysates to RNA of different compositions *ex vivo*. +, ZAP immunoprecipitated with anti-ZAP antibody; −, mock-precipitated control (IP WB). RNA was quantified by qPCR using R1 sequence-specific primers for the different mutants (UpA-H, CpG-H, UpA-L, and combined WT, CpG-L, and CpG/UpA-L; Table S2). ns, no statistically significant difference from the WT.

To more directly evaluate the propensity of ZAP to bind to RNA sequences enriched for CpG and UpA, we devised an *ex vivo* immunoprecipitation assay format in which uninfected cell lysates were incubated in a reaction mixture containing anti-ZAP antibodies and RNA transcripts of the R1 region. The ZAP/RNA complexes were subsequently immobilized, stringently washed and bound RNA quantified using primers matching the insert sequences (Fig. 5). ZAP binding to R1 RNA sequences showed a greater (100-fold) affinity for CpG- and UpA-enriched sequences, similar to what was observed for E7 containing these sequences (Fig. 4). This assay allowed us to investigate whether the lower but above-background level of ZAP binding to WT E7 sequences (compared to the no-antibody control) resulted from the presence of a lower but nonzero number of CpG and UpA dinucleotides in the sequence (*n* = 51 and 49, respectively). However, binding of ZAP was comparable to that of the zero-CpG, low-UpA, and combined zero-CpG/low-UpA sequences (Fig. 5, right). These findings suggest a level of ZAP binding to RNA sequences independent of the presence of CpG or UpA dinucleotides.

### Characterization of ZAP binding to UpA-enriched RNA sequences.

**FIG 6:**
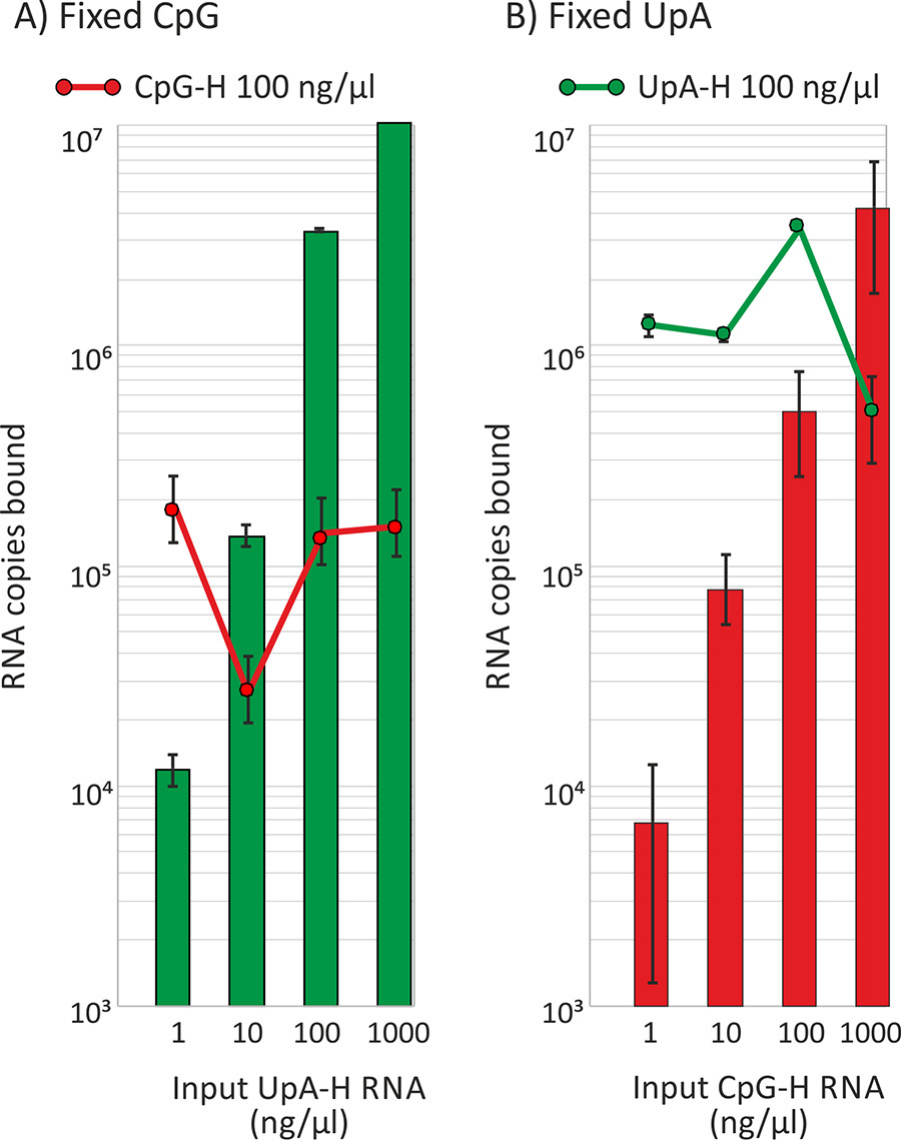
Coincubation of replicon RNA with fixed concentrations (100 μg/μl) of either (A) CpG-H or (B) UpA-H replicons in the presence of various concentration of the heterologous replicon. RNA binding was quantified by CpG- or UpA-specific PCR to enable transcripts to be differentiated (IP WB) (Fig. S4 and Table S2). Bar heights show the means for two biological repeats; error bars show SDs.

In this study (Fig. 2A) and in previous studies (1), both viruses and replicons with increased UpA numbers in either coding regions or the 3′ UTR showed ZAP-dependent attenuation. UpA-H sequences within a viral RNA transcript or a replicon (Fig. 4B) additionally showed greater binding to ZAP than the WT constructs, in both cases with greater affinity than a sequence comparably enriched for CpG. Since enrichment for CpA and UpG did not attenuate E7 replicons (Fig. 1B), it seems unlikely that ZAP binding to UpA arose simply through a reduced selectivity of the dinucleotide binding site in ZAP for YpR (i.e., any pyrimidine followed by any purine). To investigate whether CpG and UpA binding occurred through separate binding sites, we performed a competition assay in which CpG binding was assayed in the presence of differing concentrations of a UpA enriched sequence and vice versa (Fig. 6).

10.1128/mSphere.01138-20.5 FIG S3 Western blot analysis for the experiment whose results are shown in Fig. 5. Binding of ZAP in IFN-β prestimulated cell lysates to RNA of different compositions *ex vivo*. WB of input long (L) and short (S) isoforms of ZAP (~100 kDa and ~70 kDa, respectively) and GAPDH in uninfected cell lysates detected by immunostaining with anti-ZAP polyclonal antibody or GAPDH polyclonal antibody. The lower panel shows a WB for immunoprecipitated ZAP. Download FIG S3, PDF file, 0.1 MB. Copyright © 2021 Goonawardane et al. 2021 Goonawardane et al. This content is distributed under the terms of the Creative Commons Attribution 4.0 International license.

10.1128/mSphere.01138-20.6 FIG S4 Western blot analysis for the experiment whose results are shown in Fig. 6. Competition assay for CpG-H and UpA-H binding to ZAP. WB of input long (L) and short (S) isoforms of ZAP (~100 kDa and ~70 kDa, respectively) and GAPDH in uninfected cell lysates detected by immunostaining with anti-ZAP polyclonal antibody or GAPDH polyclonal antibody. The lower panel shows a WB for immunoprecipitated ZAP. Download FIG S4, PDF file, 0.1 MB. Copyright © 2021 Goonawardane et al. 2021 Goonawardane et al. This content is distributed under the terms of the Creative Commons Attribution 4.0 International license.

The binding of the CpG-H replicon was unaffected by coincubation with differing concentrations of the UpA-H replicon, even if in 10-fold molar excess. Similarly, the binding of the UpA-H replicon was unaffected by coincubation with the CpG-H replicon over the same range of relative concentrations. These findings are most consistent with the existence of separate binding sites for CpG and UpA dinucleotides in ZAP or associated, coimmunoprecipitated proteins.

**FIG 7:**
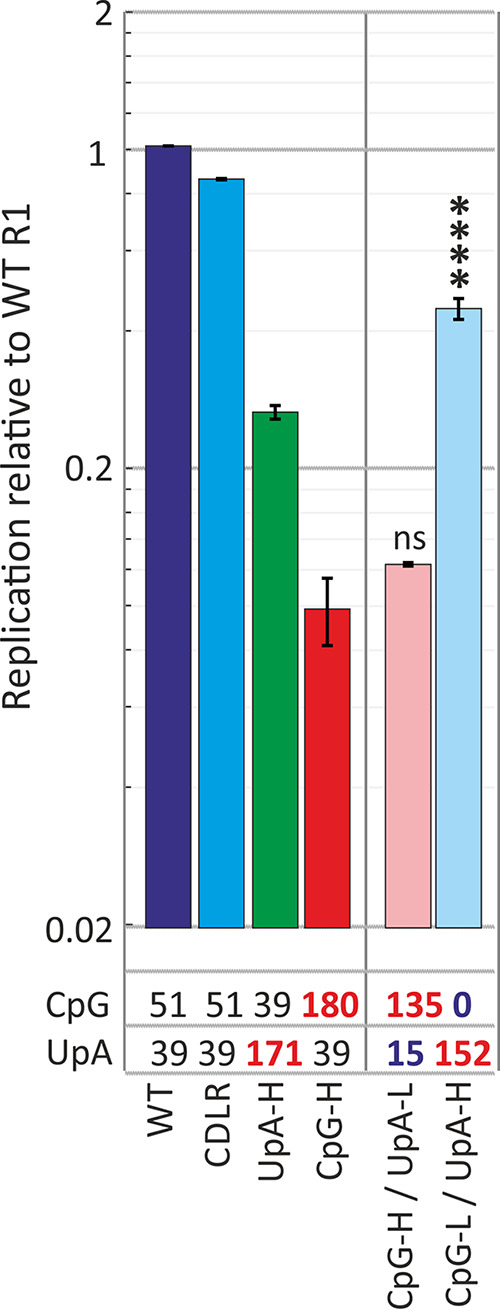
Replication of mutants of E7 replicons with minimized and maximized CpG and UpA dinucleotides. The totals of each dinucleotide in each 3′ UTR sequence are indicated under the graph; red numbers indicate totals that were deliberately maximized and minimized (for these mutants while retaining protein coding). Bar heights show the means for two biological repeats; error bars show SDs. ns, no statistically significant difference from the WT construct; ****, *P* < 0.0001.

Previous investigations of ZAP and UpA-mediated effects on E7 replication and that of other viruses have not considered whether both efficient binding and ZAP-mediated attenuation of replication requires the copresence of both CpG and UpA dinucleotides in the same RNA sequence. The observation of the potential existence of separate recognition sites does not rule out this possibility. To investigate this, we constructed a pair of replicon mutants in which the inserted 3′ UTR sequence was constructed to contain either zero CpG dinucleotides and maximized numbers of UpAs or vice versa (Fig. 7). The replication of the CpG-H/low-UpA (UpA-L) mutant was substantially attenuated despite the almost complete absence of UpA dinucleotides. Conversely, the UpA-H/zero-CpG construct was attenuated similarly to the control UpA-H mutant that possessed near-WT levels of CpG. Both mutants showed slightly less attenuation than the original mutants, although this was proportional to the lesser degree of CpG and UpA elevation possible while retaining mononucleotide frequencies and coding in these mutants. Overall, these findings show that either dinucleotide can independently attenuate replication.

### Binding of OAS3 to compositionally modified RNA.

**FIG 8:**
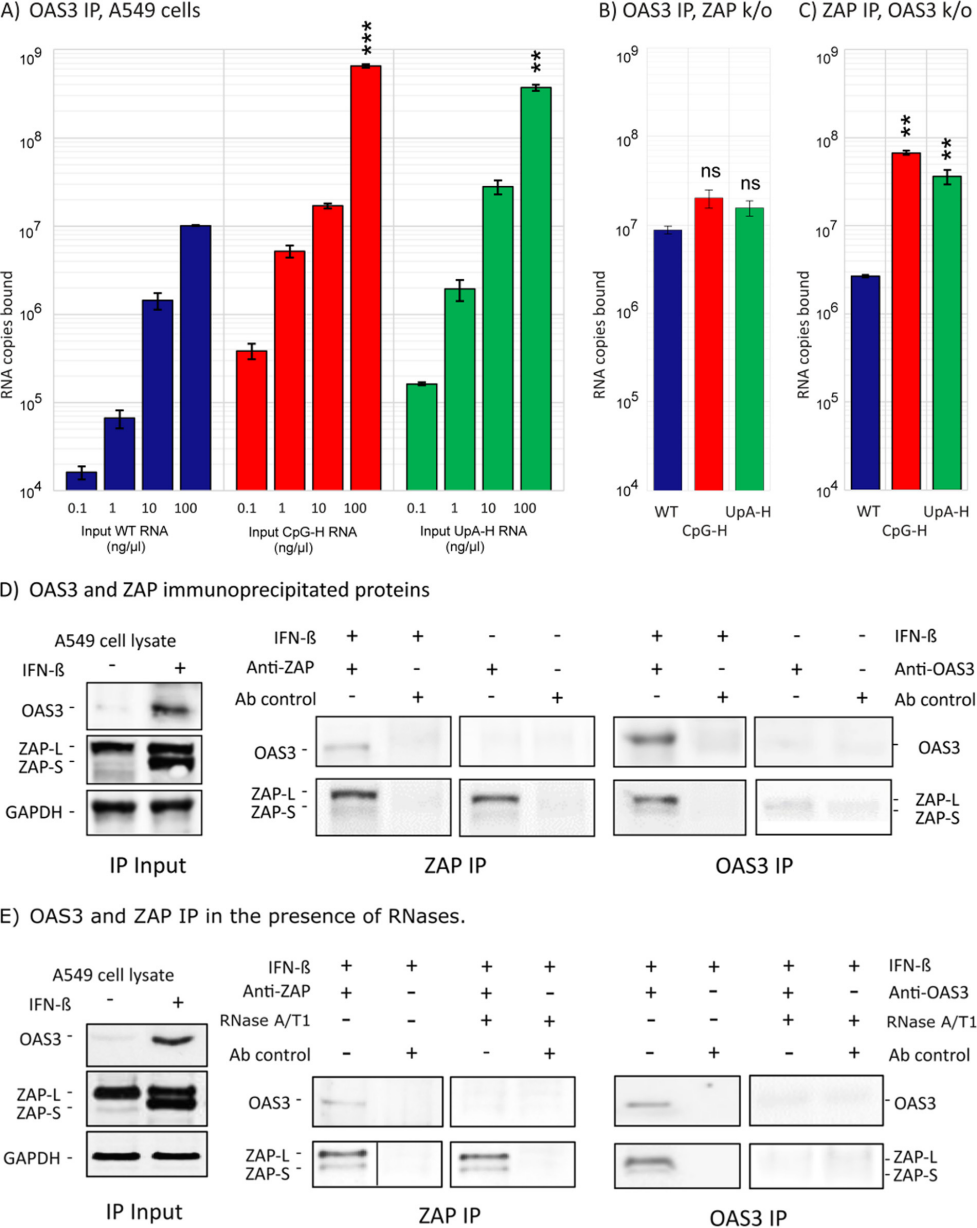
(A) Pulldown of different input quantities of E7 R1 insert RNA sequences by OAS3. Binding was quantified by qPCR. (B and C) Pulldown of 100 ng input WT, CpG-H, and UpA-H RNA transcripts in ZAP KO cells and in OAS3 KO cells, respectively. The significance of differences from WT construct is as follows: **, *P* = 0.0015; ***, *P* = 0.0002; ns, no statistically significant difference from WT. (D) Western blot detection of ZAP and OAS3 by specific antibodies in cell lysates with and without IFN-β prestimulation (left) and after immunoprecipitation by ZAP and OAS3 antibodies (center and right). In both IPs, ZAP and OAS3 (after IFN-β induction) coprecipitated, indicating their physical interaction. (E) Western blot detection of ZAP and OAS3 by specific antibodies in IFN-β prestimulation cell lysates in the presence of RNases A/T1 (left) and after immunoprecipitation by ZAP and OAS3 antibodies (center and right). In both IPs, ZAP and OAS3 coprecipitated, indicating their physical interaction.

We previously observed that expression of OAS3 was required for ZAP-mediated restriction of high-CpG and UpA mutants of E7 viruses and replicons. The nature of the interaction between ZAP and OAS3, whether direct or indirect, is unknown, although, as we reviewed, it appeared unlikely structurally that the RNA binding site in OAS3 would show any affinity for the single-stranded genomic or transcript sequences of E7 we have studied to date (1). To investigate this experimentally, we modified the *ex vivo* immunoprecipitation (IP) assay to pull out OAS3 using an anti-OAS3 specific antibody and investigated its binding to E7 R1 RNA sequences of different compositions (Fig. 8A). Surprisingly, OAS3 demonstrated a propensity to specifically bind to CpG-H and UpA-H RNA sequences comparable to that seen in the ZAP IP assay (Fig. 5).

However, the ZAP dependence of the phenomenon was demonstrated by the absence of dinucleotide-specific binding to R1 RNA in an OAS3 IP in the ZAP KO (B8) cell line (Fig. 8B), where binding affinities to the CpG-H and UpA-H transcripts were comparable to that of the WT sequence. These results indicate that RNA binding in the OAS3 IP is mediated through the coprecipitation of ZAP. Contrastingly, a degree of sequence specificity was observed in the complementary experiment, i.e., ZAP-IP of OAS3 KO cells (Fig. 8C), although immunoprecipitation was approximately 10-fold lower than in the parental A549 cells. Since ZAP expression in OAS3 KO cells was comparable to that in the parental A549 cells (1), the reduced binding did simply originate from off-target alteration in ZAP expression in the OAS3 KO cell line.

To analyze this potential cellular interaction further, we probed the OAS3 immune precipitate for ZAP, and vice versa (Fig. 8D). Cell lysates from A549 cells showed constitutive expression of the long (L) isoform of ZAP, while the expression of the short (S) isoform and of OAS3 was minimal or undetectable. Both were potently induced after pretreatment with IFN-β (Fig. 8D, left). In the absence of E7 replicon RNA, subsequent immunoprecipitation of the cell lysate with anti-ZAP antibody also immunoprecipitated OAS3 after IFN stimulation (Fig. 8D, middle). Similarly, IP with anti-OAS3 antibody coimmunoprecipitated both isoforms of ZAP (Fig. 8D, right). Next, to investigate whether ZAP-OAS3 interaction is mediated through RNA binding, we performed the same co-IP assays in the presence of RNases (Fig. 8E). The binding of ZAP to OAS3 and vice versa was undetectable upon RNase treatment, suggesting that the interaction and coimmunoprecipitation were dependent on the presence of bound RNA sequences. Their demonstrated physical association under the *ex vivo* conditions used in the IP assay indicates that apparent binding of high-CpG or high-UpA RNA in the OAS IP assay (Fig. 8A) may have been mediated by ZAP or other proteins coimmunoprecipitated in the assay (see Discussion).

**FIG 9:**
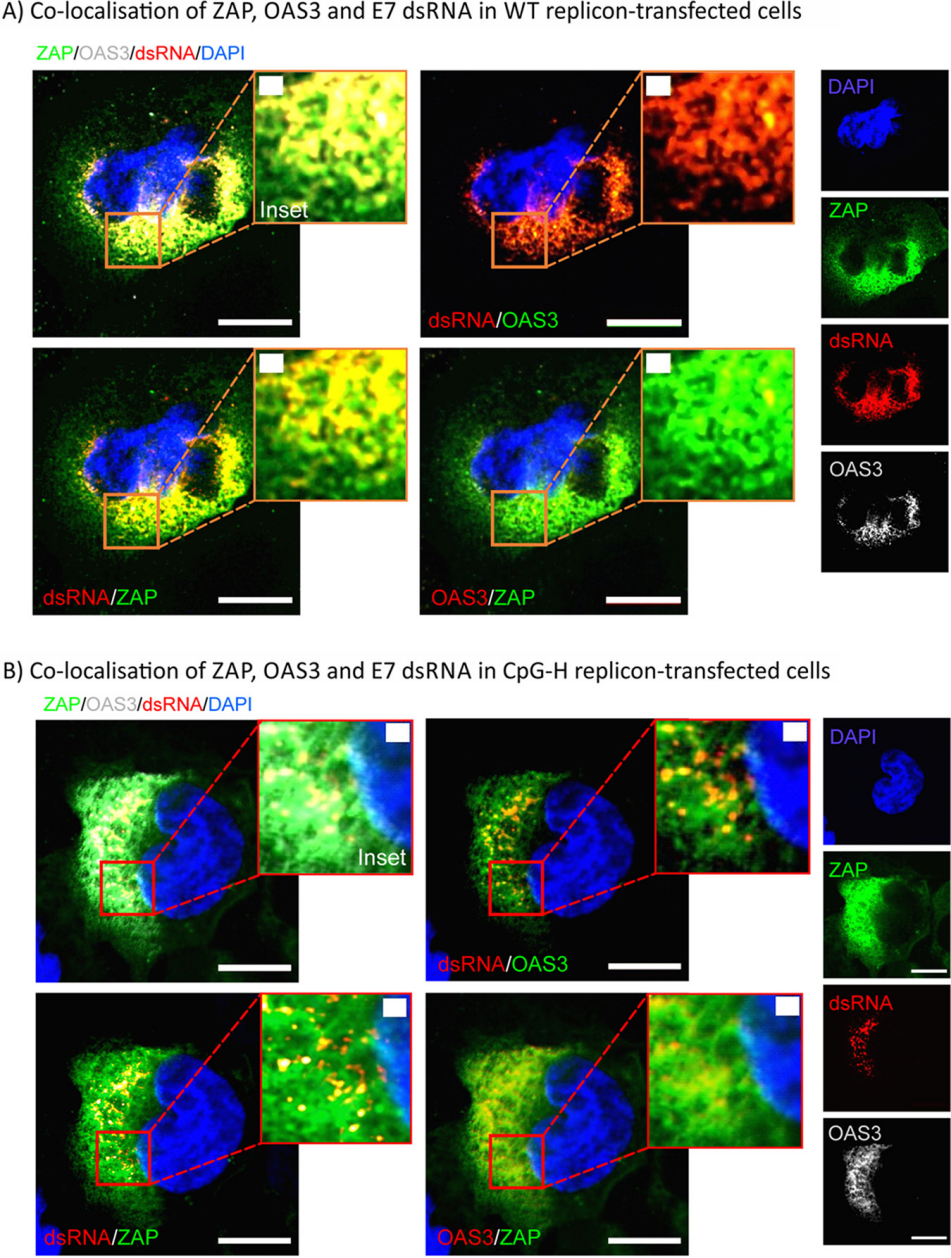

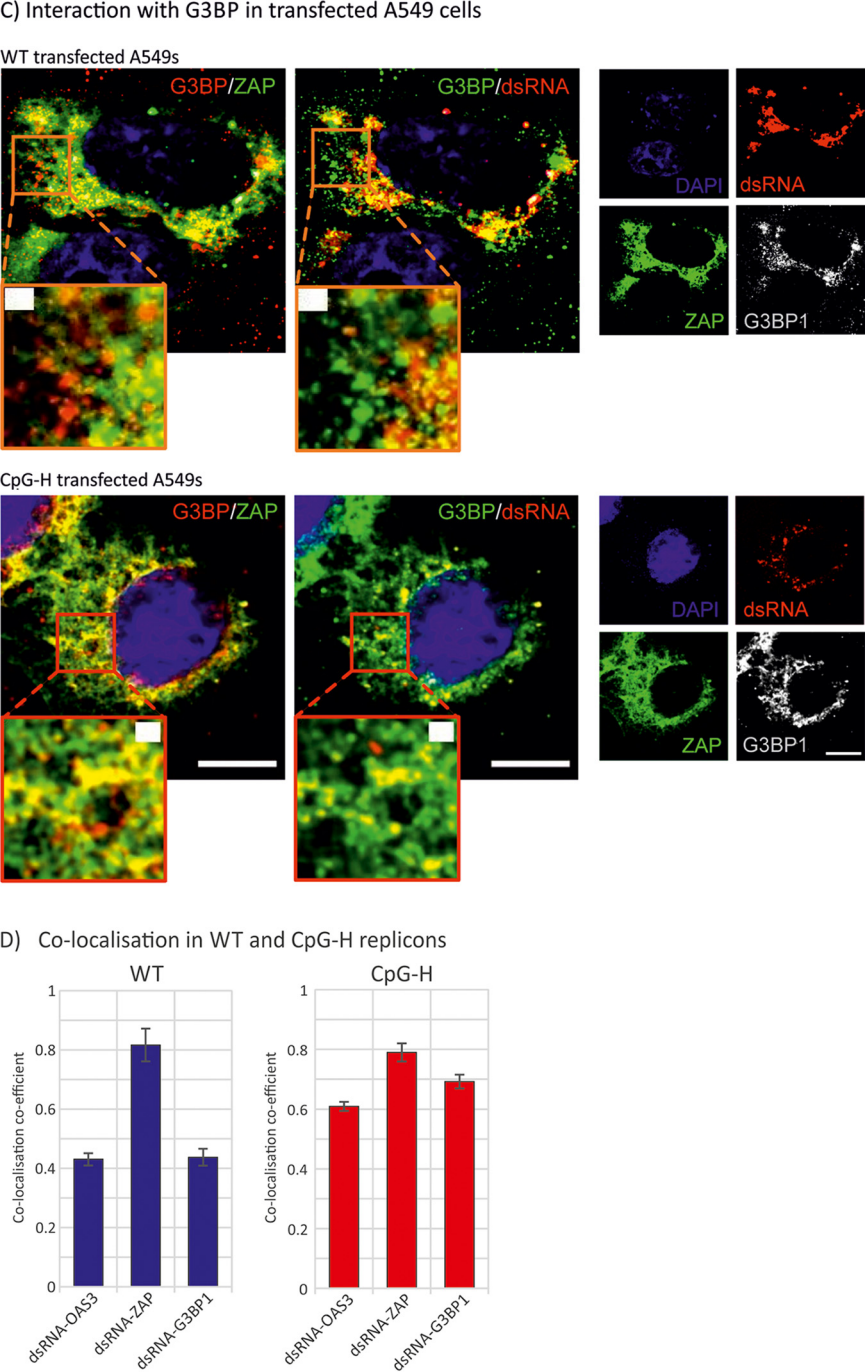
A549 cells were transfected with the (A) WT or (B) CpG-H replicon transcripts, and at 6 h p.t., cells were fixed with 4% PFA and costained with ZAP (16820-1-AP; Proteintech), OAS3 (ab188111; Abcam), and dsRNA (J2; SCICONS). Nuclear DNA was stained with DAPI. Bars, 10 μm (A) and 1 μm (B). (C) Visualization of E7 dsRNA and ZAP localization with stress granules stained by G3BP (ab220524; Abcam). (D) Quantitation of colocalization of E7 replicon (dsRNA) with ZAP, OAS3, and G3BP in WT and CpG-H replicon-transfected A549 cells using Pearson’s correlation coefficient. Bar heights represent the means for three separate cell fields; error bars show SDs.

Further evidence for the close association of ZAP and OAS3 in infected cells was provided by confocal microscopy of E7-transfected cells and immunostaining for ZAP, OAS3, and E7 (Fig. 9). An antibody to the cellular protein GTPase-activating protein-binding protein 1 (G3BP1) was included to localize stress granules and their potential colocalization with virus replication and ZAP/OAS3 (Fig. 9). Virus replication was localized by detection of cytoplasmic dsRNA by specific antibody (J2; SCICONS) in preference to our previous detection by antibodies to a viral capsid protein; the locations of assembled virions may not coincide precisely with sites of virus replication. WT and CpG-H transfected replicons induced different levels of replication at 6 h (Fig. 9A and B) but in both cases, replication complexes (red) colocalized precisely with both OAS3 and ZAP (to produce yellow dots) apart from in the immediate perinuclear region. Similarly, although localizations were more diffuse and expression levels different, ZAP colocalized with OAS3. Different combinations of ZAP, OAS3, and dsRNA were costained with G3BP1 (Fig. 9C): in particular, the amount of dsRNA colocalized with G3BP1 appears to be much higher for the CpG-H transfected A549 cells compared to WT transfected cells; a higher proportion of dsRNA did not associate with G3BP1 and led to the appearance of distinct red and green puncta. This was confirmed by quantitative analysis (Fig. 9D): for CpG-H and WT replicon-transfected A549 cells, the colocalization coefficients were 0.70 and 0.44, respectively. Similarly, substantially less colocalization of OAS3 with E7 replicon dsRNA was observed in WT E7-transfected cells.

## DISCUSSION

### The E7 replicon model.

This study used a wide variety of synthetic sequences to characterize the interaction of ZAP and associated cellular proteins to viral RNA and its association with virus attenuation. The use of a previously developed replicon enables effects of cellular restriction of replication through modification of the untranslated 3′ UTR to be uncoupled from potential compounding effects of changes in translation efficiency from altered codon usage and codon pair frequencies that might occur in compositionally modified sequences. It also enables much more extensive mutagenesis, since compositional changes do not have to be synonymous and preserve protein coding. The ability to fix G+C content in almost all mutants generated for the project (Table 1) removed a further potential confounding factor influencing replication. The attenuation achieved through modification of the 3′ UTR sequence was more restricted (approximately 20-fold for a replicon with an inserted CpG-H sequence; 4-fold for UpA-H) than that in low-MOI (multiplicity of infection) replication assays with E7 virus. However, the replicon format provides a much more direct, more quantitative, and better-normalized metric of replication ability. Investigation of the effects of compositional modification of the E7 replicon (and the virus) was additionally not compounded by potential unintended effects of sequence modifications on splicing sites and other RNA signal motifs that may be deleted or created *de novo* and compromise protein expression (6).

### Alternative dinucleotides and CpG context and attenuation/ZAP binding.

We investigated several questions regarding dinucleotide recognition and virus attenuation, for example, the possibility that attenuation might have arisen through a greater frequency of self-complementary dinucleotides in the sequence and a resulting greater degree of RNA folding as predicted by RNAFold. The observation of no attenuation and WT levels of binding to ZAP of mutants enriched for GpC and ApU (Fig. 1B) provides evidence against this possibility. Furthermore, the lack of attenuation and ZAP binding of CpA-H and UpG-H mutants indicates that ZAP binding, and its apparent affinity for both CpG and UpA dinucleotides, was not simply due to its having a broader binding specificity for any pyrimidine followed by any purine. The data showing that UpA and CpG did not compete for binding sites in ZAP (Fig. 6) and that CpG and UpA were not dependent on each other to achieve attenuation (Fig. 7) support this conclusion.

Recent studies have demonstrated that effects of additional CpG sites on attenuation of HIV-1 were dependent on genome location and positioning within genomic regions forming RNA secondary structures (6, 7). On a smaller scale, we found additionally that attenuation of replicon replication and binding to ZAP mediated through CpG dinucleotides were dependent on their immediate sequence contexts (Fig. 2 and 4), where the identities of 5′ and 3′ bases exerted a robust effect on replication attenuation and differences in affinity for ZAP binding in the IP assay. These findings suggest that there may be specifically favored bases around the CpG binding ZAP that promote binding. It is indeed intriguing, although not commented on in the studies, that both currently published structures of RNA bound to ZAP have U residues 5′ of CpG (23) or both 5′ and 3′ U sites (22); perhaps these structures were selected from other candidates because of their propensity to cocrystallize with ZAP.

From analysis of the topology and size constraints of the CpG binding site and immediate upstream and downstream bases (22, 23), it was concluded that there should be no base constraints on the base immediately 5′ (−1) of CpG and not part of the CpG binding site (23). Similarly, the topologies of both 5′ (−1) and 3′ (+1) bases were considered to not impose any base constraints. Both conclusions fail to match the experimental findings of the current study. It was further predicted that the identity of the −2 base (G) was critical for recognition (22). The ZAP structure study was published after our experimental studies were concluded, although there was, in retrospect, no association between ZAP binding affinity (or replication attenuation) and numbers of GNCG motifs in the various constructs analyzed in the current study (data not shown). The flexibility of the replicon design will, however, allow these structure-based binding predictions to be more systematically investigated in the future.

At another level, the experimental finding of strong 3′ and 5′ base context effects on CpG recognition and resulting attenuation (Fig. 2, 4, and 5) indicates a potential and more general effect of surrounding bases on CpG representation in host and viral RNA sequences. Further investigation of the representations of ACGA, CCGC, GCGG, and UCGU tetranucleotide frequencies in the human or other mammalian transcriptome compared to frequencies in nontranscribed RNA would be a good starting point, as would a more focused analysis of effects of 3′ and 5′ bases in ZAP binding assays described previously.

### The ZAP interactome.

The study provides a convincing although incomplete explanation for the previously observed codependence of ZAP and OAS3 in mediating the attenuation of CpG-H (and UpA-H) mutants of E7. OAS3, while seemingly necessary for attenuation, seems unlikely to be able to bind and discriminate between single-stranded RNA (ssRNA) sequences of different dinucleotide compositions, primarily because of its binding specificity to long dsRNA sequences (21). However, an artificial CpG-H (or UpA-H) sequence possesses elevated frequencies of self-complementary bases that make the formation of duplexed, internally base-paired stretches of RNA more likely to occur than in native sequences. However, we found that sequences comparably enriched for GpC and ApU sequences with their equally overrepresented frequencies of self-complementary bases showed no attenuating effect (Fig. 1B) and no greater-than-WT levels of binding to ZAP (Fig. 4).

Indeed, the findings in the current study provided evidence for a cooperative interaction between the two proteins, through their close physical association through their coprecipitation (Fig. 8D), the dependence on ZAP for RNA binding in the OAS3 binding assay (Fig. 8B), and colocalization of ZAP and OAS3 (and replicon dsRNA) in infected cells (Fig. 9). ZAP has indeed been shown to colocalize with stress granules (SGs) and other cytoplasmic structures (15, 16, 29, 30) and shows a dependence on other proteins, such as TRIM25 and the nuclease KHNYN, for its antiviral activity against HIV-1 (6, 8). However, the 78 cellular proteins, many associated with stress granules, that were verified to co-IP with ZAP on mass spectrometry analysis (15) indicate its much larger interactome and intimate connection with other antiviral, stress response, and RNA degradation pathways. Among the latter, co-IP and in some cases direct binding with ZAP were observed for the 3′-5′ exosome component EXOSC8 (RRP43), EXOSC5 (RRP46), the helicase DHX30, and the 5′-3′ exoribonuclease 2 (XRN2). While TRIM25 was identified in that list, KHNYN and OAS3 were not, despite the clear evidence for their coprecipitation in targeted Western blotting (WB) assays (6) (Fig. 8D). We further demonstrated that coprecipitation of ZAP and OAS3 was dependent on the presence of high-CpG or high-UpA RNA sequences (Fig. 8E). This dependency matches the previous report that the ZAP antiviral activity is mediated through RNA-dependent interactions with TRIM25 via its PRY/SPRY domain (31).

This study provides further evidence for the attenuating effects of elevated frequencies of the UpA dinucleotide on E7 replication and binding in ZAP and OAS3 IP assays (Fig. 1B, 3, 6, and 7). These observations contrast with predictions from recent structural studies that UpA could not be accommodated within the ZAP binding site as an alternative to CpG (22, 23). The possible existence of an entirely separate UpA binding site on ZAP or associated protein was indeed experimentally supported by the observed absence of binding of alternative YpR dinucleotides (Fig. 1B) and the lack of competition by CpG- and UpA-enriched RNA sequences for binding sites in competition assays (Fig. 6). It is conceivable that the complex of ZAP-associated proteins identified by IP may include one or more further RNA binding proteins that could mediate the apparently specific recognition of UpA-enriched sequences, including those with zinc finger domains in the ZAP interactome (15) such as zinc finger RNA-binding protein (32). However, the possible existence of an alternative UpA recognition protein in the larger stress granule/ZAP complex does not square with the apparent dependence on ZAP expression in the OAS3 IP assay (Fig. 8B). In the ZAP KO (B8) cell line, only WT background levels of binding were observed for the UpA-H (and CpG-H) transcript, which contrasts with the 2-log-greater binding in ZAP-expressing A549 cells (Fig. 8A).

There are undoubtedly vast complexities in the cellular response to RNA sequences of different compositions and configurations. For example, ZAP binding may directly or indirectly activate OAS3 independently of RNase L; this might represent a complementary or additional effector pathway to KHNYN, perhaps targeting RNA in the different cellular compartments of E7 in replication complexes and HIV-1 sequences expressed as mRNAs. The use of a range of different viral models with their different replication strategies is clearly essential to fully understand the nature and purpose of CpG- and UpA-mediated restriction of virus replication in mammalian cells.

## MATERIALS AND METHODS

### Cells, viruses, and reagents.

Cells were maintained in Dulbecco’s modified Eagle medium (DMEM) (Life Technologies) supplemented with 10% heat-inactivated fetal calf serum (FCS) (Gibco), 100 U/ml penicillin, and 100 μg/ml streptomycin (Life Technologies). A549 ZAP knockout (KO) and A549 OAS3 KO cell lines with deletion of all three alleles were grown as described previously (1).

### Construction of E7 replicons and ncR1 expression plasmids.

The design and construction of the E7 isolate Wallace infectious clone and replicon with various nucleotide compositions in R1 were described previously (20, 33). Briefly, the R1 region (nucleotides [nt] 1878 to 3119) in the viral coding sequence of the full-length E7 cDNA clone pT7:E7 was selected for mutagenesis. R1 is 1,242 bases in length and extends from VP3 to part of VP1 in the capsid domain. The mutated version of R1 included sequences permuted in base order by the algorithm CDLR in the SSE 1.3 package (34). This retained the coding and mono- and dinucleotide frequencies of the original sequence. Other mutants were high-UpA (UpA-H) and high-CpG (CpG-H) mutants in which dinucleotide frequencies were maximized while protein coding was retained. R1 sequences were cloned into the 3′ noncoding region of E7 via flanking SalI/HpaI sites, and correct insertions were confirmed by sequencing (Table 1). For the replicon, the structural genes of E7 were replaced with a firefly luciferase gene with zero CpGs and lowered UpA frequencies and cloned in the pRiboT7 vector.

The R1 sequence was further altered such that it contained the same number of CpG (*n* = 51) and UpA (*n* = 62) dinucleotides as the WT R1 sequence, but the context of the CpG dinucleotides was altered. Four mutants were created where an A, C, G, or T base was placed immediately 5′ and 3′ of each CpG dinucleotide in the sequence, creating the ACGA, CCGC, GCGG, and UCGU motifs (Table 1). Mutagenesis preserved mononucleotide frequencies identical to those of the native sequence, although the degree of mutagenesis was too great to maintain protein coding. To control for the latter difference from the WT sequence, a fifth mutant was made in which the bases 5′ and 3′ of each CpG dinucleotide were randomized. A second set of five high-CpG context mutants was made which contained 102 dinucleotides instead of 51. Additional mutants with retained mononucleotide frequencies, noncoding but with incorporation of elevated frequencies of a range of other dinucleotides, were inserted into the replicon. All R1 insert sequences used in the study are provided in FASTA format (Table S1). RNA preparation and assessment of replication ability were performed as previously described (1). Quantitative real-time PCR (qRT-PCR), SDS-PAGE, and immunoblotting were performed as previously described (1).

10.1128/mSphere.01138-20.1 TABLE S1 Composition of synthetic ncR1 insert sequences. Download Table S1, DOCX file, 0.03 MB. Copyright © 2021 Goonawardane et al. 2021 Goonawardane et al. This content is distributed under the terms of the Creative Commons Attribution 4.0 International license.

10.1128/mSphere.01138-20.2 TABLE S2 Primers used for E7 RNA quantitation. Download Table S2, DOCX file, 0.01 MB. Copyright © 2021 Goonawardane et al. 2021 Goonawardane et al. This content is distributed under the terms of the Creative Commons Attribution 4.0 International license.

### Replicon RNA luciferase assay—RNA preparation.

The replicon was further modified by insertion of modified sequences in the nontranslated 3′ UTR as described in Table 1. Replicon plasmids were linearized using NotI, and RNA transcripts were synthesized *in vitro* using T7 RNA polymerase (MEGAscript T7; Thermo Fisher Scientific) for 4 h. pTK-Ren (Promega) expressing *Renilla* luciferase was cotransfected as a transfection control. PTK-Ren was linearized using XbaI. RNA transcripts were DNase treated (Thermo Fisher Scientific) and cleaned with a RNA Clean and Concentrator column (Qiagen). RNA integrity was confirmed on agarose gels, and the concentration was determined with Qubit fluorometric quantitation (Thermo Fisher Scientific).

Cells were seeded at 2 × 10^4^ cells per well in 96-well plates and transfected with 50 ng of replicon and 10 ng of pTK-Ren using the transfection reagent Lipofectamine 2000 (Thermo Fisher Scientific). At the 6 h posttransfection, cells were washed with phosphate-buffered saline (PBS) and harvested in passive lysis buffer (Promega), and the luciferase activities were measured using the dual luciferase reagent kit and GloMax multidetection system (Promega). The firefly luciferase readings were normalized to the *Renilla* value for each sample. In some formats investigating effects of gene KO, luciferase expression was further normalized to that of the parental WT A549 cell line control.

### Immunofluorescence.

IF assays were performed as previously described (1), using cells seeded onto 19-mm glass coverslips in 24-well plates. At 6 h posttransfection, cells were fixed in 4% paraformaldehyde (PFA) and permeabilized with 0.1% (vol/vol) Triton X-100 (Sigma-Aldrich) in PBS for 15 min. Coverslips were washed in PBS and blocked in blocking buffer (3% [wt/vol] bovine serum albumin [BSA] in PBS) and the primary antibody applied at the relevant dilution in 1% (wt/vol) BSA (Sigma) in PBS and incubated overnight at 4^0^C. To remove any unbound primary antibody, cells were washed three times in PBS before the application of the relevant Alexa Fluor 488-, 594-, or 647-conjugated secondary antibodies (Life Technology) diluted 1:750 in 1% (wt/vol) BSA in PBS, followed by 2 h of incubation at room temperature in the dark. DAPI (4′,6-diamidino-2-phenylindole) staining and microscopy were performed as previously described (1). Cytoplasmic colocalization of dsRNA with OAS3, ZAP, or G3BP1 was estimated through Pearson’s correlation coefficient (*R*) by the PSC colocalization plug-in (ImageJ, NIH) (35). *R* ranges between −1 (perfect negative correlation) and +1 (perfect positive correlation), with 0 corresponding to no correlation. Colocalization calculations were performed on >10 cells from at least two independent experiments.

### Quantitative real-time PCR.

To quantify viral RNA copy numbers, total RNA was extracted from cell supernatants with an RNeasy kit (Qiagen). The amount of viral RNA for the given time point was determined against a standard curve of quantified transcript RNAs using the Quantitect RNA kit (Qiagen) and primers annealing specifically to the 5′ UTR of E7 (Table S2). qPCRs were performed using a StepOne plus real-time PCR system (Applied Biosystems).

Total cellular RNA was isolated using an RNeasy kit (Qiagen). About 40 ng RNA was used as the template for qRT-PCR using Superscript III (Invitrogen) and Fast SYBR green master mix (Applied Biosystems). The abundance of the target mRNA was normalized to that of the GAPDH housekeeping gene coamplified by qPCR (primers are listed in Table S1). To quantify viral RNA copy numbers, total RNA was extracted from cell supernatants with a NucliSENS EasyMAG system (bioMérieux). The amount of viral RNA for each time point was determined against a standard curve of quantified transcript RNAs using the Quantitect RNA kit (Qiagen) and primers annealing specifically to the 5′ UTR of E7 (Table S1). qPCRs were performed using a StepOne Plus real-time PCR system (Applied Biosystems).

### RNA-binding protein immunoprecipitation.

IP in the previously used intracellular format was performed as previously described (1). For the *ex vivo* assay format, cell lysates were first prepared from monolayer cultures with or without beta interferon (IFN-β) (eBioscience) stimulation (100 ng/ml) 24 h before harvesting in 15-cm dishes using radioimmunoprecipitation (RIP) lysis buffer containing protease and RNase inhibitors (Millipore). Cell lysate and RNA transcript (at the indicated concentrations of 100 ng/μl for RIP and 1, 10, 100, and 1,000 ng/μl for competitive RNA binding) mixtures for each reaction were then incubated with magnetic A/G beads associated with either rabbit anti-ZAP (5 μg per reaction; Proteintech) or normal rabbit IgG (Millipore) as described above. For RNase treatment, after washing, beads were resuspended in extraction buffer supplemented with the RNase A/T1 mix (Thermo Scientific no. EN0551) for 20 min at room temperature with shaking at 1,000 rpm. The beads were then washed four times with RIP lysis buffer, and the immune complexes were eluted by boiling in Laemmli sample buffer for 5 min at 95°C; the proteins were analyzed by Western blotting.

For the competitive RNA binding assay, four binding assays were conducted for each experiment. Cell lysates were incubated with CpG-H E7 transcripts at 1:0.1, 1:1, 1:10, and 1:100 molar mass ratios to UpA-H and vice versa (UpA-H E7 transcripts at the indicated molar mass ratios to CpG-H). Cell lysates were incubated for 5 h, and the samples were washed six times, with complete resuspension of beads between washes and 15 min incubation with rotation for the last two washes. After washings, the samples were resuspended in RIP wash buffer and divided into aliquots. A 100-μl aliquot of immunocomplexes was reserved for analysis of the protein fraction. After beads were immobilized, the wash media were discarded, and beads were resuspended in Laemmli sample buffer. About 10 μl of the inputs was also mixed with Laemmli buffer. All the samples were heated for 5 min at 95°C and separated by SDS-PAGE for Western blot analysis. In parallel, viral RNA were purified from another aliquot of immune complexes and inputs, using the RNA viral kit (Zymo Research). RNAs were eluted in 15 μl of RNase- and DNase-free water. The copy number of E7 present in each fraction was determined by qPCR as described above.

### SDS-PAGE and immunoblotting.

Following separation by 4% to 15% SDS-PAGE, proteins were transferred to a nitrocellulose membrane using a semidry transfer unit (Bio-Rad). The membrane was blocked in blocking solution containing PBS with 0.1% Tween and 5% skimmed milk (Sigma) and immunoblotted with the following primary antibodies: ZCCHV (for ZAP) (Abcam no. ab154680) and anti-GAPDH (Abcam no. ab8245). Antibody binding was detected by horseradish peroxidase (HRP)-conjugated secondary antibodies followed by chemiluminescence detection by the ECL Prime Western blotting reagent (GE Healthcare).

### Statistical analysis.

Statistical significance was determined in GraphPad Prism using either Student's *t* test with Welch’s correction or an ordinary one-way analysis of variance (ANOVA) with multiple comparisons using Bonferroni's correction.
