# Supplementary figures and images for: Association of Zinc Finger Antiviral Protein Binding to Viral Genomic RNA with Attenuation of Replication of Echovirus 7
Source: mSphere. 2021 Jan 6;6(1):e01138-20. doi: 10.1128/mSphere.01138-20 (PMC7845596; doi:10.1128/mSphere.01138-20)

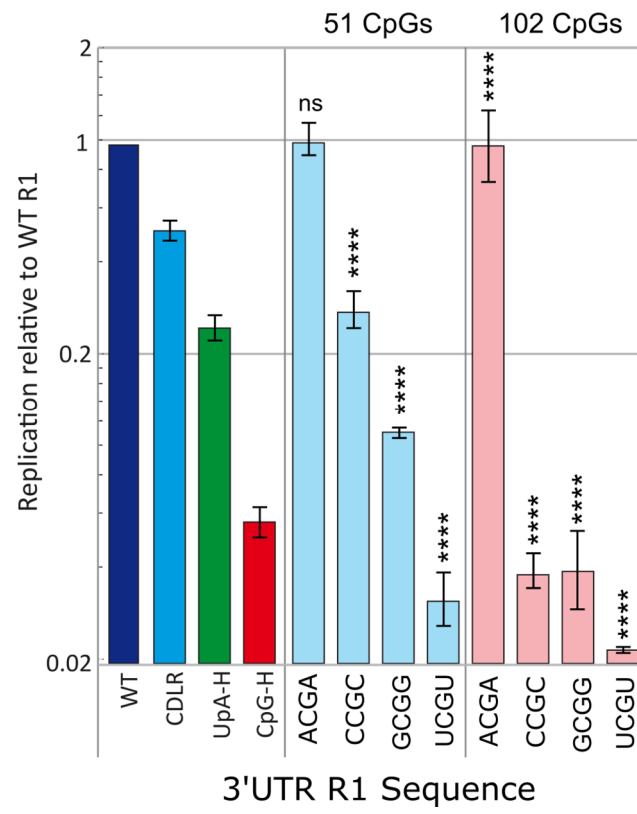

Supplement: FIG S1 [file mSphere.01138-20_sf001.pdf]

A) CpG-H R1 RNA transcript

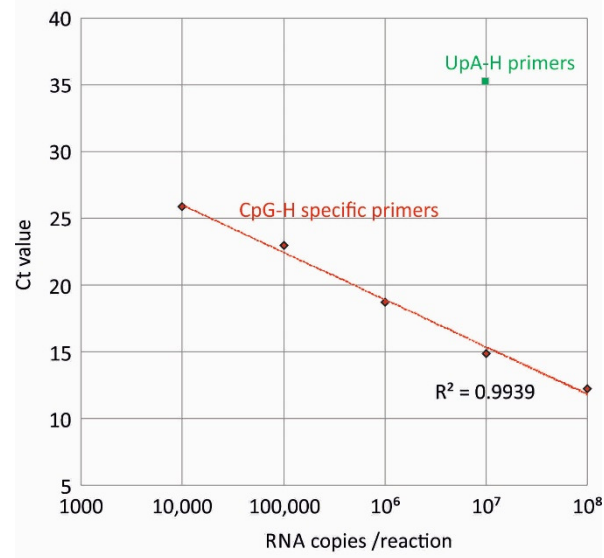

B) UpA-H R1 RNA transcript

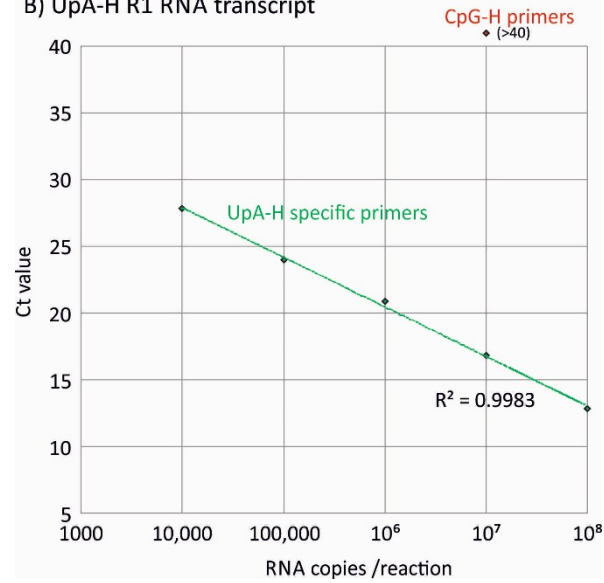

Supplement: FIG S2 [file mSphere.01138-20_sf002.pdf]

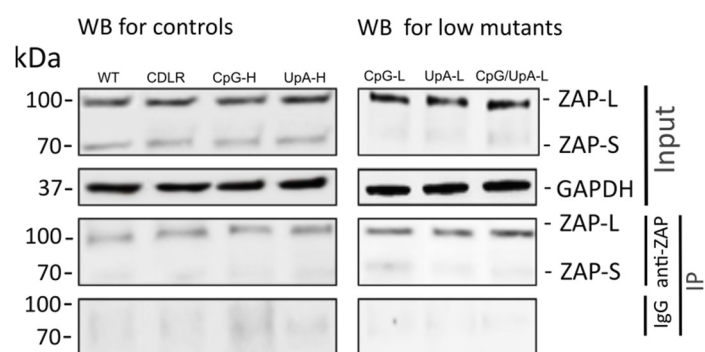

Supplement: FIG S3 [file mSphere.01138-20_sf003.pdf]

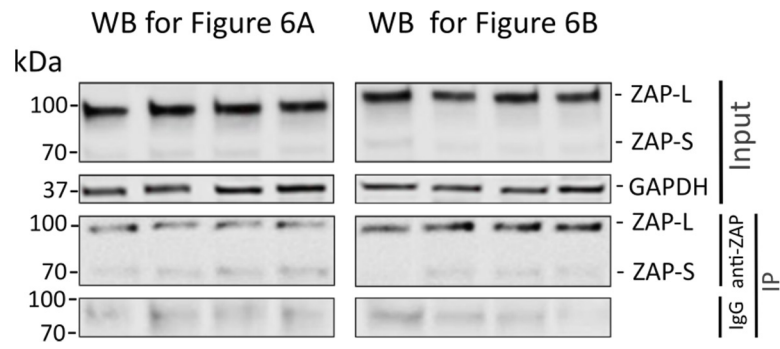

Supplement: FIG S4 [file mSphere.01138-20_sf004.pdf]
