# Supplementary material for: Association of Zinc Finger Antiviral Protein Binding to Viral Genomic RNA with Attenuation of Replication of Echovirus 7
Source: mSphere. 2021 Jan 6;6(1):e01138-20. doi: 10.1128/mSphere.01138-20 (PMC7845596; doi:10.1128/mSphere.01138-20)
Supplement: TABLE S1 [file mSphere.01138-20_st001.docx]

TABLE S1

Composition of synthetic ncR1 insert sequences

>Echo7_wt

TCCGTGGTGCCCGTCAACAATATCAAAGTCAACCTGCAAAGCATGGATGC

GTATCATATTGAGGTCAATACCGGGAACCACCAGGGGGAAAAGATTTTTG

CGTTCCAAATGCAGCCGGGGTTAGAGTCTGTTTTCAAGAGAACCCTTATG

GGGGAGATTCTTAATTATTATGCACACTGGTCAGGGAGCATTAAGCTGAC

ATTCACATTTTGTGGATCGGCGATGGCAACTGGAAAACTCTTGTTAGCGT

ATTCACCACCAGGTGCTGATGTGCCCGCGACCAGGAAACAGGCGATGTTA

GGCACACACATGATTTGGGATATCGGGCTTCAGTCGAGCTGTGTTTTGTG

CATCCCATGGATAAGTCAGACACACTACCGGTTAGTGCAACAAGATGAAT

ACACGAGTGCAGGCAATGTGACGTGTTGGTACCAAACAGGAATAGTGGTG

CCCCCTGGCACTCCAAATAAGTGTGTAGTGCTTTGTTTTGCATCAGCTTG

TAATGATTTCTCAGTTCGAATGCTTAGGGACACCCCTTTCATCGGACAAA

CAGCACTGCTGCAAGGCGACACCGAAACGGCTATTGACAATGCAATCGCC

AGGGTAGCAGATACGGTGGCGAGCGGTCCTAGTAATTCGACCAGTATCCC

AGCACTCACAGCAGTTGAGACAGGTCACACGTCACAAGTCGAGCCCAGCG

ATACAATGCAGACTAGACATGTCAAAAACTACCACTCGCGTTCTGAGTCA

ACCGTGGAAAACTTTCTAAGTCGCTCCGCTTGTGTGTACATCGAAGAGTA

CTACACCAAGGACCAAGACAATGTTAATAGGTACATGTCGTGGACAATAA

ATGCCAGAAGAATGGTGCAATTGAGGAGAAAGTTTGAGCTGTTTACATAC

ATGAGATTTGATATGGAAATCACGTTTGTAATCACAAGTAGACAACTACC

TGGGACTAGCATAGCACAAGATATGCCGCCACTCACCCACCAGATCATGT

ACATACCACCAGGTGGCCCGGTACCAAACAGCGTAACAGATTTTGCGTGG

CAGACATCAACAAACCCCAGCATTTTCTGGACAGAAGGAAACGCGCCACC

TCGCATGTCTATTCCATTCATCAGTATTGGCAATGCATATAGCAACTTCT

ATGACGGGTGGTCACACTTTTCCCAAAACGGTGTGTACGGATACAACGCC

CTGAACAACATGGGCAAGCTGTACGCACGTCAT

>E7_R1_CpGH_UpAL

TCCGTCGTTCCCGTCAACAACATCAAAGTCAACTTGCAAAGCATGGACGC

GTATCACATCGAGGTCAACACCGGGAACCACCAGGGCGAAAAGATTTTCG

CGTTCCAAATGCAGCCGGGTTTGGAGTCGGTTTTCAAGCGAACGTTGATG

GGCGAGATTCTCAATTATTACGCGCACTGGTCGGGGAGCATCAAACTGAC

GTTCACGTTTTGCGGATCGGCGATGGCGACGGGAAAACTTTTGTTGGCGT

ATTCGCCGCCGGGCGCCGACGTTCCCGCGACGCGGAAACAGGCGATGTTG

GGCACGCACATGATTTGGGACATCGGTCTTCAATCGAGCTGCGTTTTGTG

CATCCCGTGGATCAGTCAGACGCACTACCGATTGGTGCAACAAGACGAAT

ACACGAGCGCCGGAAACGTGACGTGTTGGTATCAAACCGGAATCGTCGTT

CCGCCCGGAACGCCGAACAAGTGCGTCGTTCTTTGTTTCGCGTCCGCGTG

CAACGATTTCTCCGTTCGAATGCTTCGCGACACGCCGTTCATCGGACAAA

CGGCGTTGTTGCAAGGCGACACCGAAACGGCGATCGACAACGCGATCGCG

CGCGTCGCCGACACGGTCGCGAGCGGTCCGAGCAATTCGACGAGCATTCC

GGCGCTCACCGCCGTCGAGACGGGTCACACGTCGCAAGTCGAGCCGAGCG

ACACGATGCAGACGCGACACGTGAAAAACTATCACTCGCGATCGGAGTCA

ACCGTCGAAAACTTTTTGAGTCGCTCCGCGTGCGTTTACATCGAAGAGTA

CTACACGAAGGACCAAGACAACGTGAATCGTTACATGTCGTGGACGATCA

ACGCGCGACGAATGGTTCAATTGCGTCGAAAGTTCGAGCTTTTCACGTAC

ATGCGATTCGACATGGAAATCACGTTCGTCATCACGAGCCGACAACTTCC

CGGGACGAGCATCGCGCAAGACATGCCGCCGCTCACGCATCAGATCATGT

ACATTCCGCCCGGCGGACCGGTTCCGAACAGCGTGACGGATTTCGCGTGG

CAGACATCGACGAACCCGAGCATTTTCTGGACCGAAGGAAACGCGCCGCC

GCGCATGTCGATTCCGTTCATCAGCATCGGCAACGCGTACAGCAACTTCT

ACGACGGTTGGTCGCACTTTTCGCAAAACGGCGTTTACGGATACAACGCG

CTGAACAACATGGGCAAGTTGTACGCGCGTCAT

>E7_R1_CpGL/UpAH

TCTGTAGTACCAGTTAATAATATTAAGGTCAACCTACAAAGCATGGATGC

TTATCATATTGAGGTTAATACAGGTAACCACCAGGGGGAAAAGATATTTG

CCTTCCAGATGCAGCCTGGGCTAGAGTCTGTGTTTAAGAGAACCCTAATG

GGGGAGATACTAAACTATTATGCACACTGGTCAGGTAGTATAAAGCTTAC

ATTTACATTTTGTGGATCAGCTATGGCTACTGGTAAACTGTTACTAGCAT

ACTCACCACCAGGGGCTGATGTACCTGCTACTAGGAAACAGGCTATGCTA

GGCACACATATGATATGGGATATAGGGCTACAGTCTAGCTGTGTACTATG

TATACCCTGGATTAGCCAGACACACTATAGGCTAGTACAACAAGATGAGT

ATACTAGTGCAGGTAATGTAACCTGTTGGTACCAGACAGGTATAGTAGTA

CCCCCTGGCACCCCTAATAAGTGTGTAGTACTATGTTTTGCATCAGCCTG

TAATGATTTCTCTGTAAGGATGCTAAGGGATACCCCCTTTATAGGGCAAA

CAGCACTACTACAAGGAGATACTGAAACAGCTATAGATAATGCAATAGCC

AGGGTAGCAGATACTGTAGCTAGTGGGCCTAGTAATTCCACCAGTATACC

AGCTCTTACAGCAGTAGAGACAGGGCACACATCACAGGTAGAGCCCAGTG

ATACTATGCAGACTAGACATGTTAAAAACTACCACTCTAGATCTGAGTCT

ACTGTAGAGAACTTTCTTAGTAGATCTGCCTGTGTATATATAGAGGAGTA

CTATACTAAGGACCAAGATAATGTAAATAGGTATATGTCATGGACTATTA

ATGCCAGAAGAATGGTACAGTTAAGGAGAAAGTTTGAGCTATTTACATAT

ATGAGGTTTGATATGGAGATAACATTTGTTATTACTAGTAGACAACTACC

TGGTACTAGTATAGCACAAGATATGCCCCCACTTACCCACCAGATTATGT

ATATACCACCTGGGGGCCCTGTACCAAATAGTGTTACAGACTTTGCTTGG

CAGACATCTACTAACCCTAGTATATTCTGGACAGAAGGTAATGCCCCACC

TAGGATGTCTATACCATTTATAAGTATAGGTAATGCTTATAGTAACTTCT

ATGATGGGTGGTCCCACTTCTCCCAGAATGGGGTATATGGCTATAATGCC

CTTAATAATATGGGTAAGCTATATGCTAGACAT

>UpG-Max

GTCCGCTGCTATATACGTGAAACACAGCACTACATGATCATACACCTAAC

ATGTGCGTGTACAAATGCCCGTGCTGTGTTTGGTGGGTCAAATGTGACAT

TGTCTACAAGGTGAATGCATGGTGCTGGTGAGCATTGCATACGATTCACT

GACGGGCACTGTGTTGATGTGCAAACACACACGCTGGGAGTGACACACAC

CATGTAACATGCGAACACACCGTGCTATCACAAATATTGTAAGTACCCAT

GGAGCCTTCCAGCTGCTATGTGACGATAATAGTGTGTGAACAGTGCTGTT

GTGTGCTGAGGCCTAAAGAACAAACACGTGTGTAATAGTACATGCTATAC

CCGATGCAGCACAAGCGTGACCCCATATGAAGCACGAACGTGCGAGCACT

CCTATGCAAGTTGTGCCTCATGAACCACACATGTGCAACTCACAATGCTT

GCACCTATGCCGTGCACATAACACTGCCACTGAACCATGGTATAACAGCA

TGCATGGAACCAAATGATGTGCACATGGACTGCAACACGACGTATAACCA

AAACTGCTGATGCTACACATGCAATACGACCATGCATGACGATGCGATCC

TGGTGCCACTTTAGTGGTAACGCACGAGGCCAACGGCTGACATGCAAATA

CCGACTTACACTTTACTGACAACAACGGCGTACGTAAGTATCATGACATA

CATGTGCATGAGTTGATGGGACCTGTGCTGACCACGTGAAATATGCACTG

CTGCTATGGTGTATGCCTGCAAAGCATGTGTTTCAATGTGAATGGATGAT

CACCGCCGCCACACTCAGACTAACGTGTCCAATGTGTGACACGTGTAATG

GCAATGATTGTGTGCCACTGCAAATTGCATGGACAATGCGCACACCGTGT

GTTGACGTGGAATGACCACACTATGTGCAGAAACATGATGACCACCATGT

ACCGAGTACATAATTAATCCTAGTTGTGACATAAAACTTCGACATGCTGC

GTGCTGTACACACCAACTGTCTAACTAATTGCCGCAAGCTTGTAGATGTA

CGTGGCGAAAATGCAACTGACAGGGTGACGTACACGACTGATGACATGGG

GGATTATGAGAGAATGTGCGCAAGCAATGCCTGCACACATGAATGATGTG

CACGCAATGTTGTGAGCTGCGCATGCGGCTGCACTCGGCAACTTGATACG

AGGGATCCAAAATTGTCACAATGAACTGTGCAT

>CpA-Max

CACACATATGCACAGGTTATGTCGCAGCCAGAGTGGCAATTTACATTGCA

CAATTCGTTAAGCGCAGGCGCCACAGCAGGCCATATTCAACAGGTCCATC

AATAGACGGACAGGAATGACAGCATGCAGCGGTCATACAAGGTATGCAGT

ACCAACAACATGGCCCTGATCAGCGCGTAGGTAGTTTTATTGTTCAAGGG

ACACACTCATTTGCGACACGCCATAACAGTTGCCCGTGTGTCATTCATGC

ACACACATGCAACATCCCAATCCGTTTAATGGCTCATGGTGGTGATTTAA

GTGTGGCACATATTCATGCATTTGGATCGGCACGCACGGCACAGTGGCGG

ATGCCTTTGGCAAAGCACCGGTAGCACACATTTATCACGTCACGTGCAGT

TGGTGTGCGAGTGGAAGTGGGGTTGACACATAGATCATTGGAGAAAGCAG

CGCACATAATCAGCATACAGACCGGTACGGATACATGTAACATTATGCAT

ACCACACATATAGATCATATACAGTGATCAAAATTGGCATGCATGTCGCG

ACAATGCCACATCACACAGTCAGCGCACATCAGTAGGAAGGAAGCAGACA

CATTTTTAGCAGTATCGCATCACAGGGACAAGGCGCGCACGGCCCAATGG

ACAGGTAGATTGATGTGCATTTTACGCACATCCATGCAGTATTGTCATTG

GTCGCAGTGTGTTAGACACAACAACAGACATTTGAACGATACAGTGCATC

GTGTCAGTGCACGGGGTTCACGGCCTGCAGATCTAATGCACAGAGGTATC

ACATACCGCCATCGTGGACAGGTCACATAAGCAGCATCCACAACAGTTTG

GCTCGTGGCACATAGCGTGATGAGAGTGGCAAAATACAAATTTGCACAAC

AATGCATTAGCAGCAGATCTCATTGCACGCAGACGGTCGTCCTTGCACGT

CACGCATAGTTGGTCATTGCACAGCAGACTACAGTGTGGAGCAAGGCTGG

AAACAAACACATGGAATCACACATTGGTTATGCTATATTTCATGACGGTT

AGTATATCATTATACTTTTGGTCGTCACACATCACACGTCCCTATTGCAC

ATGTTACATAGCAGATTAGCAGTTCTTGAGCAGTGCATCATCGTAGACTT

GCATTGTTAGCTTACACCCAGAACTGGCACACAAATGGCGTTGGGTGCAG

CAAGTCATGCGATGGTCCATCAGCATGATTGGA

>UpA-Max

TATTGCCGCCACGGGTACCTATATACAGACAGTAGTCGAAGTAGGTATAA

CCTACGTAGATATGCAGCACGTTAGTACATGCCACACAGCTGTGTTACGT

AGTATATAGTATACCATGCAGGTAGGCTGGTAACCGAGTTACGAGGTACA

GTCATGCCTTAGCCCTCGCGAATGCATGGTAGTAATCATACCTAGCACTG

CGCATTAACACAGCTGCAGCAGGATATATGTATAGCAGTACGTGCAGCCC

ACCAACCACTTACATGCCCACCAGCCACATACACTGCCGCAGGCAATAAT

AGTAGCCGCGCTATGTATGCATACCATGCAATGGAGCACTACTAGAATGC

AATATGTTATAACGTACGTATATACACATGGGACACTGGCTTGGCTGCAC

AGTAGGGTGTGTGCGCCCGTGGGCCTACTGACAACTGCATATAAATAGAA

TGTATTAACATAGTATACGTGCTCGCGCATGCTGTGCAGTGACTATGTAG

CATATATACATAGGTGGCCATGTGCTAGTATACCGCATATGGTATACAGA

TATTATGCTGCAGGCAGTATGCTATGCAGGGGCAGACACACGCATGAAGC

TGGCATACCATACATATACAGGGTAATGTAGGGCGCGCAGGCTAATACAC

CGCCCATGCAGGGGTCAATCTAGGCATAGATCGCATGTTATATAGCCGCT

ACCAGTGGTATTATTTATAGATACCAGCAACGTATGTGTATGCATATGTA

GAATGATAGTTTGCACCATGTTGGCCTGCGCTCTACTACCAGGCGTAACG

TACTGAACAGGCACAGGGTACCAAATACACTGCAGGCGCAGTACGCGCCA

CTACGTAGCAGCACTAGCTCGGTATGTATGTGAATGGGGGGAGCCACACC

CGGTGTGTACCCAACACGCATATGTAGGCTACACAGCAGTAACTGCGTAC

TGCCACATAGTACCTGCGTGCATATACCTGTAGTGCACTGCATGGGCAGG

AGTGTCTAATATGTATATGTATAATTCAGCTGCAAGTCGCATTAGACCCC

AATGTGCATACTAAGGTTATGTACAACACGCACGTACAGCAGACTAACAG

ATGCATAACATTAGCAGCATACGTATAACATAACAGGCTCATACTTACAG

TATGAGTAGCGGTAGCAACACGCGGTATACCCACGAGTGCCCCACTAGAC

ATGCAGGGGCTGTAGCCTACGGTATACATCAGT

>CpG-Max

CGAACGGCGAGTGAGACAAGCGATATCATGTGCATGATATGAGACGGTCT

TCGCATGTTACGTTGGTTCAGTGCCAAGCGAATACACGCAGATCGCACGC

GATGATCAACAAATCATACGACAAAATTCACACGCGTACGGGGTATCACG

ATCGCAAGTGCGTGCGACAAATAATCGCCAGGTATATAACGCGTGTTATG

ATGAACCGCAGGTGTCACAAGAAACGCATTTTCGTGCAATGATGTTGATA

CACGATTGTCGCATGCGCACATTACATGCCGACACATTCAAGATCGGAAC

GGCGACGGCACTGCGTTGGCATTGACACGATGCACGAAGCGTACAACGTG

AAGTACGGTGCGCGGCATGTCGAATCGTCACGTCGCAGATCGATCACCGA

CATCAAGTCCATCATCAGAGCAGGTCCGCAATCGTGTATGTCTCGATCAA

TCCATGTCATTGCATCTTTAACAAACACACGCACGTCGTATACGGGTGGC

ACAATCAATTGAATGTTGACGAAAACATCACGCATTCCATGCCGGCATGC

AACATTTGCCCATGGCGCGTCGTCAAATAAACCCGAGGCGCATTGTGATC

AATCCAAGACGCACAATGCTGTTCGTTTCGTTGTGACGAATTAACGATGG

GCGTATGCGAACATATTGCGGTGCGACGCGTGCACGCATATACGTATAAT

TTCGCTCACACGTTTGCACTTACGATTAAATGCACATTGTGCGCCGCCGA

CGTGAACACACGTTGTGTCGTTCATCGAACGTTGTGTGCATGCGAATGCG

TTGCGGGTCTCACGGATGTGAGATTACGTAGAATAAATTCGTCACGGGAT

CGCAGGATGTCATACGCGTGACCGTCGAACCGTACAGGCATTAATTGTAT

CATAATGAAGCACGCAACAGCGTAAAGCATCAATCATTGACGATGCATAG

CGGATTACGTAACGGATCACATACACCGAATCGATGCATATTCATGCACA

AGGTACGTTTGATCACGTAAATATTAACGGGCTAAAGACAACGATTGACG

TGCGCGCATCACATACGTACGATGATCACATTTGCGCGCATGTAAAATCT

GGCGCACAGCATCATACATCAGATATCGATATCATTGAACAAATGTAGCA

AATCGCGGTCATACACGGAGTGTCTGCATGACAATAATCCATGCGTAAGT

ATTGCGACGATTACATCGCAATACGTGTTTGAG

>ApU-Max

GCCAGGGAGCCGAATATCCCATAATCATTCGACCTGCATTGCATGGGGGA

TATGATATACGATATCAATTCCGGATATCGACATGGATGACTGATACATG

CGGGCCACATGCAGCCGGGGGGTGTGATATACTTAATGGATAATCACACG

AAGGAAGAAATAGATCGATTGGCACTCAAGTCAGGGGGATGCATGCGGAA

TATCATATGGGGATGGATGGATGATGCTAGATATCAAATACCAATCGCGG

AACATCAGCATTGAGCTGATGATCCCGACCGCCTGGACCGGGCGGTGATA

TATCAATCCAGGATGTGATCCTCCGGGCGGCAGAATGGCCGAGATGATTG

CAGGAATAGATCTCATCCGAATTCACATCATGGAAAGCCCCCAGATGATG

ACTCGGAGGCCATCTATGGGAATGGTGATGATCATACAAATATCGTATGG

CCTCCTGATCTTACCTGTTGGAAGATTGAGCATCGTCAGGCATCATCAAT

GAATGATCCCACATGATGAATGCATGGGGCCAACATAATCCAATGTTCCA

TGATCAGGCAGCATGGATCATTCGGAATGGCTGAAGACACATCCATCAAG

AGGATGTCAGATTCGGCTGCGGGATGGCCCAATCAATCGAATAACATCAT

ATTTATCATCTCATCCGCGATTGGGCCCGCGCCAAAATGCGCGCCATGAT

GCTATATGCAGATATTACATGAATCTCACCACCCATCGATCGCAGTGGCA

GCATATGAAGGCTAATCGAGACCTAATCCATGAATGGATATCGAAATGAT

CAATCCCCTGGACCCCATCCAAGATATATGGGAAATGATAAGGATAGTGA

TGGCATGATACCGGGGGCCAATGATGATCCATGATGAGCAATGGATGATA

TGGATCATGGACACGGCCTATCATATTATAATCATATTACGTCGTCAGAA

TGGGCCCATCATTATCACGATGCAGATGCCATCCACCATATTGATCAAAG

ATTAATCTCCAGGGGGCCCATTTAAGATCTGCGGCTCATTTGGGGCGATG

CCGGATCCATCCACTCCAATCCATTCCATGGAGTTATGCCAGGCGATCAT

CCGCAAGCCCCCCATATTCATCATGCATCTCATACCAGGATGCTAATCAT

ACATCATGTGAGCACCCAAATATCCATTCGGGGGGTCCATGATCATCGCC

CTGATATACCTGGGCATTCCAATCGCACGGCAT

>GpC--Max

TTTTTGCTGCGCCTCCACCGCCGAAAATGGAAGCTGCAAACAATGCATGC

GCAGCATATTCATATGCATAAACAGGAGCAACAAAGCGAAAAACTTTTTG

CGTTGAAAATGTACCCGATGTGTTCATGTCGCTTCAAGAGCAGCATTATA

GCTGGCAGCAGCAATTGCTGCTGCGAGTGGGCCGGCAGCATTAAGGCAAG

CTGCACAGCTTGGCAATCAGCACATCAAGCTGAACCACTTTTGTTAGCGT

ATTGAGCAGCAGCTGATTATATGCTAAATCACAAAAGCGCGACGCTCTTA

AGGCGCTAGCTGATGCAGGCTATCTCCCTTAAACAGCATTTTTGCTTTTG

CATGGCATGGCTAAGTGAGGCAAAGCAAATAGCAGCTGCAGCGGCTCTGT

ATAATGCTGCAGTCGCTTTCCTGGCTTTGGCAGCAAAACAAATACTGCTG

TGCACTGAAAAGCTGCATAAGTTTGTAATTATTTCTTTTTGATAAGCTTT

TAATGATTTATGCGCCGGAATGGCTAAAAAGCTGCGTTTGCTAGCAGCAA

AAGCAATGGCTCAAGAGAGCAGCTGCAAGTCTGTTGCTAATGCGCTAAGC

AGAATAGCAAAGCCGCTTTGCAGCGCTGCTAGGCGCTTGAGCAGGATGCC

ACCACTGCAAGCTCTTGAGAGAGCTGCAAGCTGCGCGCTTTAGGCCGCAC

ATAGAATGAACAATAGCGATTCCAAAGCGTGCCAGACTTTTGCTGAGCAA

ACGCTTCAAAACTTTCTAACGTTGCTCGCTTTGCTGCACATTGCCGAATA

AGAGCCCAAGGAGCAATCAAATGCTAATACTTACATGCGCTACACAATAA

ATGTGACAGCAATGCTGAAATTGACGCAAAGCCGCGACCTCGCTAGATAG

ATGATATTGCATATGAAAGGCACGCTTCTGCTGGCGCCTGCGGCAATAAC

GCAGGCTATTAAAAAAGCAGCTATGCCGCTGTGCGCACAACCTATCATCT

AAATGCAAAGCTGCTGGGCAGTAAGAAAGCTCATAGCAGGCCTTGTGTGG

CAGAGATCAAGCAAGCTTAGCATTTGCGCCAAACAGCGGAACCAGCCAGC

TCGAATGGCTATTCCATGCAGCAATATGCCGAATGCATGCAGGCACTTCT

ATAGGCATTGAGCACAATTTTGTCAAGCCAGCACGCACGAAGGCAGCCAA

TTCAAGGCAATTTGCAAGTATTAAGGGCGGCCT

>UpA-Max

GCCTAGTTGGAGGCACCGCGCATAGTAGGCAACGAGGTATACTTAATAAG

AGTCTAATTCTACTCTATACCAGGCCTAATCCGCACATACATAGTATAGT

ACATATACCCGGTAAGGGGTAGAGGGGATATCAACCCGCACCTACTAGGG

GTAGGGAGCCTATACACCCCGGCTATATACCGGTAGTTCCGGGGTCAGTA

CGCCTAGCAGATCGCGGCGATTTAGCTCGCGAACTCTATACTAGGTATAT

ACTGGTACCGATTGAGTCTCGGCTACTACCCCACCCTCCCCTAGTGTACT

GTCTAATACCCCGACTAAGATGCTGCTAAGCTGGTCCATACAGTGTAGGT

AGGCTAGTACCGCCCTCACATATATTAGTCAGGGCCGTGTAAACTAGGTA

AGTAAAGCCACTCATACAGTACCACTTAAGCCTAACGGCGCTAGTAGGCA

TATGGGACAAACCCGGTAGAGAGTACGTTATACATTGACCCTAACGCCGT

AAGTCGTACCTATTTACTAAGGCGAGGTAACTTTGCAACTGTACAAAAGT

ACGCTATACAGCGTATATATATCTAGTAGCTAAGGTAGGTATACTCAGTA

CTGTATAGGGAAGGTGCCCGTCCTGGCCTTGTCAGCTATACCAGTAACCG

GGTAGTAGAAGCTATAGGTAACGTCCCAGGTAGACATATAGTAGAGTATA

CCGCCTAGCCAGTACTGAGGATAAGCTAGGTCTACCTAGACAGGTATACC

TACTAGCTATACTCTATACAGTACGCCGTGTCTAGGCAAGTAGAGTAGTA

CAACGGGGGGCAGCCCTTAGGGGGCACAGCCCGCTAGGCGGTACCAGGGT

ATAGTTCAGGCTAACGGAGTGTATAGGGGCGGTCAGCCAGAACGGACACC

TCTATAGAGCCCCCTAAATAGTGTAATCCCTGATAGGAATATAGTAGGCA

GTAACCTAGGTATACCAGCTAAAGGCGATACTAGGTACTAATATACTAGG

CCCTTATATATAATATACGAGACCTAGTACGCGAACGACCTACATAAGCC

CCTATATACCTAGGCCTAGCCTATATGGGGAGTAGGGTACCATATAGGGG

GTATATATAGGGCCTACTACCGGGGCTACATACGTCTAGAAACCATAACT

AGGTAGGACTACTACTCTAGCCTACTACAAGCACGGAGCTATACCTCGCT

CTTTATGCAAAGGTAGCCAATAGTGTATATATA

>CpG-Max

TGGATCAAGTCATAAAGGAATATAAGAATCGTTTTAACGATGACGAATCG

AATTCATACGAACCTTAACGCTTCACGCGAAGCGGGTTGAAAACGTCGCA

AGTTTCCGGTACCGTTTGTTCCGCGATGAAGGTTTTATAAAACGTCGATC

CGAAAGACGCCGACCGATTTTTACACGAGTTTATACGCGACGAAGGGACC

ATTGCCATCGTCGCGATAAATGAACAACGTTTGAAACGTCGTCGTACCAT

ATACACAATGATTCGTTGATTTCAAGGATTACGTGCGAGCGTCGATTTTC

GCGGACGACGTCGTTTGGTATATTGCGTCATGATCGAAACGGGAAAGCTG

ACGCCGTTTCATACTTCGCCGACAATTTCGCGTCGTGAAAAATTATTCGC

GATCAACGCGGTGCAATTCGGACACTTCGTATGAAAAACTCGAACGTTCG

ATCCATCCGCGCGGAAATCGCTGTTTACGTTTTCCCGTCTACGCGACTTT

TCGCGATTCGTCACTTCGAATGCGTTCGCGTTCCGCTTTCGTCGAACTCG

CGTAATTGCGTTACAGAGGAATCGAAACAAGAACGTATTGTAGAATCTGA

ACGTCGGCAACGAACGGATAACCGTTTGGTATAAACGAGATCAATATCAC

AGCACGCAACTCAAGTAGCGAAAATGAAAGGCTGTACGTCCACTACAAGA

ATCGAACGACGGCGACGGACGTTACGAACGCGAACTCCGCTCAACGGTCG

AACGACGGCCGAATTGTACGCGGTTGGTGCGTCGTTTACCGAAGAAAACC

GTACCGAATTCGCGCGAAGCGTTCACGTAAATAACGCTTTTGCCTCGTAA

ATGAAAAATTACGGGTGAACCGAACGACGAAGTTCGAGCGAGCGAAGTAA

AAGCGACCGCATTCGTAGACGGGCGTTATAATAACGATTATCGTGCGAAT

CGGTCGTCGGATTCGCGAAAATATCTTCCGATTCAACGAAAAAAAAACGA

CTTTACGAACCGATGCGACCATAACAACGAAGGCTTCATTTTTCGATTCC

TAAATACGAAACGCACTACGAATCGAATTCCGCTGCCGAAAAGTTACCGC

GCCGAAACGTATCGACGCGATCACGAGGCACGAGCGGCGATAAACGGTCG

ATCGCCGTACTCGACAATAATTTCGAGGCTCGACGTACTCATGCGGCTTG

CTCGAGCGAATGGAGAATTTCTGGATAATTCGT

>ACGA_GpG-H

GACGATTTTGACGAGAATCATAAGCCTCCTCATCTGACCTGCCACGAACG

ATATCATAGCCCTGAGTTTAACGATGGCTCTCTTGGGTGCCATGAGCTCA

CGATGTGTCTCATCACGAGGCTACACACGACTTGAGGACGAACGAATATG

CTGGTTCCTGCTACTTACTAACGAACGATTGACGACTGTCCTACTGCCAG

TGTGACGATTGACGACACGAACGAGACGATCCCACTCATCCAGGTAACGA

TAGCCACGACAATTCACGAACGACACGAACGAACGATCTTCACGACTTTA

GGGACGAATTGGAAGTGAGTTAACGAGTGCAGCACGAAAGGAGGTTTTCC

TTTCACGATGTTAGCTGTTGGTCTCTAACGATTAGGCACAAGCCAGCTGT

AGACGACTGGTCCTCCCAAGACGAGGTTGTAGGGTTTTTGACTAACGAAA

ACGAACGAGACGACCAGTATGATTGTAATTCTTTTTGACGAGACGATTGC

TAACGACTGTGCAGAACGAGATTGTAACGAACAGGACGACAACGAGTTGC

TGCATCCTGCCTCTCACGACAACGACACGAATAACGATGTGACGAACGAA

CGATTATGTTTTAACGAGACGAACGAATCTATTATGACGAACGATAGCCA

CGATGTCCCCCTGATGCCAACTGTGCAGACGAGGCATCACGACACGAACG

ATACACTGCCAAGTATGGAGTCAGCTGGTTATTGCACGACCTGACGAGAG

CACGAACGATCACTTGTATCACGACACGATGACGATTATGACGACCTGTA

GTAGACGAGCACAAGTTTTGACGATATTATTTATCAGACGATGACGATAC

ACGATTTCACGAGGAATTTTTGACGATTCCCTGACGAAGCTTTTATTTAG

GGACGAGTTGATAGTGGCCTGACGATCTTAGGAACGAGTACTGGTCTAAC

GAACTCTACAATATTGGGCCCTAAAACGAGAGTGACGATGGCCATGCCTT

ACTTAGGTGGAGTTGGGACGATATCACTGACGATATGAGTGCTCACGAAC

TTTGGCACGATTCAAACGACAGATGCTGCCCACGACTGCCACGACCTTCC

ACGATCTGTTATCACGATCCTTTGTAACGAGAACGATTATATTGTGCTGT

ACCACGAAGTCACGATTGCAACGAGGGACGACTTGTAACGATAACACGAC

ATCAGGTGGTCCTCTCAGCTCTAACGAACGATG

>CCGC_GpG-H

ACCGCTTTGACCGCAATTGATATGAGAAAAATTTTTGATTGAACCGCCGC

ATAGAGTAAAAATTGTTTTACCGCAGGTTGATTTGATGATGTGAGAAATC

CGCAGTTGAAAAATCCGCTGATAAAACCGCATGATGACCGCCCGCTTAGA

AAAGTTTTGAATAGATATTACCGCCCGCAGTCCGCTTGAAATAATGAAAA

TTTGCCGCAAACCGCACCGCCCGCACCGCAGAAAATTTTGAAAATACCGC

TAAGACCGCAAGTTTCCGCCCGCACCGCCCGCCCGCAAAAACCGCTTTTA

TGACCGCTTGGAAATGGGTGTACCGCAAAGGGGCCGCAAATGAGATTTGA

ATTTCCGCAGGTAAGATTGTGAATTTACCGCTTAGAAAAGGAAAGAGTTT

ATCCGCAGGAAAAAAAATGACCGCAGTGGTAGGTTTTTGAAATACCGCTG

CCGCCCGCACCGCAGAGTAATTTTGTAAATTGTTTGACCGCTCCGCTTGA

TACCGCAAATGAATGCCGCATTGATACCGCAAATTCCGCTGCCGCAAAAA

AATTTTTGAATGAATCCGCTTCCGCCCCGCATACCGCAAAACCGCCCGCC

CGCATATTTTTTACCGCTCCGCCCGCTGATAATAAACCGCCCGCTAAGAC

CGCAGTGAATGATTGAAAGATTGAAAGGCCGCAAAAGACCGCACCGCCCG

CTAATTGAAAAGTTAGGATGAAGATGATGTATGAACCGCATGTCCGCTGG

ACCGCCCGCAAGTTTTTAGTCCGCACCGCATCCGCTTATGCCGCAGAATA

GTAGCCGCAAAGAAGTTGTTCCGCTATTATGTAATGGCCGCATCCGCTAT

CCGCAGAACCGCAGGTTTGTTTCCGCTGAAAAACCGCAATTTTTAGATAG

GGCCGCTTGAGTATGGAAATGCCGCAGATAAAACCGCATAGTGAGATACC

GCAAAGTAATTTAGGGAAAATTAGACCGCAGTTGCCGCAGAAGGATTTGT

AAGTATGGAGTTGGATGCCGCTAGATGTGCCGCTAATTGAGAGACCGCTT

TGGAGACCGCAAATGCCGCAATGGGGAGAATCCGCAGAAACCGCTTTTGA

CCGCATGAATAATCCGCTTGAAAATACCGCATCCGCTTATATTTGAAAAT

AATCCGCTGATCCGCTTGATCCGCTGTCCGCAGTTTACCGCTAGACCGCT

GATGGGTTGAGAAATGAAATGTACCGCCCGCAG

>GCGG_GpG-H

AGCGGTTTCAGCGGAATTCATATCAAAAAAATTTTTCATTCAAGCGGGCG

GTACACTAAAAATTCTTTTAGCGGCCATTTTCAAATTCATCTCACAAATG

CGGCATTCAAAAATGCGGTCATAAAAGCGGATCATCAGCGGGCGGTTACA

AAACTTTTCAATACATATTAGCGGGCGGCATGCGGTTCAAATAATCAAAA

TTTTGCGGAAAGCGGAGCGGGCGGAGCGGACAAAATTTTCAAAATAGCGG

TAACAGCGGAACTTTGCGGGCGGTGCGGGCGGGCGGAAAAAGCGGTTTTA

TCAGCGGTTCCAAATCCCTCTAGCGGAAACCATGCGGAAATCACATTTCA

ATTTGCGGCACTAACATTCTCAATTTAGCGGTTACAAAACCAAACACTTT

ATGCGGCCAAAAAAAAATCAGCGGCATCCTACCTTTTTCAAATAGCGGTT

GCGGGCGGAGCGGCAACTAATTTTCTAAATTCTTTCAGCGGTGCGGTTCA

TAGCGGAAATCAATTGCGGATTCATAGCGGAAATTGCGGTTGCGGAAAAA

AATTTTTCAATCAATGCGGTTGCGGAGCGGATAGCGGAAAAGCGGGCGGG

CGGATATTTTTTAGCGGTGCGGGCGGTCATAATAAAGCGGGCGGTAACAG

CGGCATCAATCATTCAAACATTCAAACAGCGGAAAACAGCGGAGCGGGCG

GTAATTCAAAACTTACCATCAACATCATCTATCAAGCGGATCTGCGGCTC

AGCGGGCGGAACTTTTTACTGCGGGGCGGATGCGGCTATTGCGGCAAATA

CTAAGCGGAAACAACTTCTTGCGGTATTATCTAATCAGCGGATGCGGTAT

GCGGCAAAGCGGCACTTTCTTTGCGGTCAAAAAGCGGAATTTTTACATAC

CTGCGGTTCACTATCCAAATGGCGGACATAAAAGCGGATACTCACATAGC

GGAAACTAATTTACCCAAAATTACAGCGGCATTGGCGGCAAATCATTTCT

AACTATCCACTTCCATTGCGGTACATCTGGCGGTAATTCACACAGCGGTT

TCCACAGCGGAAATGGCGGAATCCCCACAATGCGGCAAAAGCGGTTTTCA

GCGGATCAATAAGGCGGAAAATTCTAGCGGTTGCGGTTATACAAAAAATT

ATTGCGGATTTGCGGCATCTGCGGTCAGCGGTTCATAGCGGTATTGCGGA

TCTCTTCACAAATCAAATCACTAGCGGGCGGTT

>UCGU_GpG-H

GTCGTCAAAGTCGTCAGCTGTAAGACAAAGAAGGGGGAACCAATCGTTCG

TTACACTACAAAGAGAGGTATCGTGCAACACAGGGAAGGAGCCCAGAAGT

CGTCAACAGGAAAGTCGTGGATACCATCGTCAACACCTCGTTCGTCTACA

AAACACAAGCATAACTAATATCGTTCGTGCTTCGTTTCAAATAAGAAAAA

GACCTCGTCAATCGTCTCGTTCGTGTCGTCAAAAAGCAGAGAAATATCGT

TACACTCGTGAAAGGTCGTTCGTCTCGTTCGTTCGTCACAATCGTGAGTA

GGCTCGTGACCCAAACCCCTTATCGTCAAACCCTCGTCAAGACCAGGGAA

AGAGTCGTGCATACACAGGCAACAATATCGTGTAGCAAAAGGAAACACCT

ACTCGTGAAAGAAGAAAGACTCGTCAAACTACCCAGGAGCAATATCGTGA

TCGTTCGTCTCGTCAACTAAGAGGGTAAAGAGCCCAATCGTGTCGTGAAG

TATCGTGTGAAACAGTCGTCATGCTATCGTAAAATTCGTGCTCGTGAAGA

TCCAGGAACAAGCAGTCGTCATCGTGTCGTGAATCGTCAAATCGTTCGTT

CGTCTACAGGGTATCGTGTCGTTCGTGAATAGTACATCGTTCGTTAAACT

CGTCATGACACAGAGCAAACAGAGACACTCGTCAAAACTCGTGTCGTTCG

TTAGAAGGAAAAGTAGCCAAGATCAGCCATAGGCATCGTGAACTCGTGCA

GTCGTTCGTCAAAGGATAGGTCGTGTCGTGGTCGTCTACCTCGTCAGGTA

ATATTCGTGCAAGAGGGAACTCGTTAATAGGTAGGACTCGTGATCGTTAA

TCGTGACATCGTCAACCCTGGGTCGTGAAGAAATCGTCAAAGGTAGATAC

ACTCGTCCACATATCACAAAATCGTCACTAAAATCGTGTAACTGGATATC

GTGGGATAAAGTAGGGCCCAGTAGGTCGTCACAGTCGTCCCAAACCAGGT

AAGTAGGGCCACCACCATCGTTACCCAGATCGTTAGCAGGCACATCGTCA

GGGCCATCGTCAAAGTCGTCAAAGCCCCACCTCGTGACCATCGTGAAAAG

TCGTCTGCATAGATCGTGATCTTCTATCGTTTTCGTGTATACCCACAGGT

AGATCGTCAAGTCGTGGGAGTCGTGTGTCGTGAGGTATCGTTAGATCGTC

AACCCCACACAAGGGAAGACATATCGTTCGTCC

>XCGY_CpG-H

TCCGATTTTGCCGAGAATCATAAGCCTCCTCATCTGACCTGCCTCGATCG

GTATCATAGCCCTGAGTTTACCGGTGGCTCTCTTGGGTGCCATGAGCTCA

CGTTGTGTCTCATCCCGGGGCTACACTCGGCTTGAGGGCGAACGCATATG

CTGGTTCCTGCTACTTACTATCGAGCGATTGACGTCTGTCCTACTGCCAG

TGTGACGTTTGGCGACTCGGGCGAGGCGATCCCACTCATCCAGGTAGCGT

TAGCCCCGACAATTCATGGTCGGCCCGACCGAACGCTCTTCACAACTTTA

GGGACGCATTGGAAGTGACTTATCGGGTGCAGCTCGAAAGGAGGTTTTCC

TTTCCCGTTGTTAGCTGTTGGTCTCTACCGGTTAGGCACAAGCCAGCTGT

AGGCGACTGGTCCTCCCAAGACGTGGTTGTAGGGTTTTTGACTAACGGAA

CCGACCGGGACGCCCAGTATGATTGTAATTCTTTTTGTCGAGTCGCTTGC

TAGCGACTGTGCAGATCGAGATTGTAGCGAACAGGCCGTCATCGGGTTGC

TGCATCCTGCCTCTCTCGACACCGACACGGATATCGATGTGGCGATCGCC

CGGTTATGTTTTAACGTGGCGAGCGAATCTATTATGGCGACCGGTAGCCA

CGCTGTCCCCCTGATGCCAACTGTGCAGACGTGGCATCTCGACCCGAGCG

ATACACTGCCAAGTATGGAGTCAGCTGGTTATTGCACGTCCTGTCGGGAG

CCCGTGCGATCACTTGTATCTCGCCCCGGTGGCGTTTATGTCGGCCTGTA

GTAGACGCGCACAAGTTTTGACGTTATTATTTATCAGACGTTGACGGTAC

ACGCTTTCGCGTGGAATTTTTGGCGATTCCCTGTCGAAGCTTTTATTTAG

GGGCGAGTTGATAGTGGCCTGACGTTCTTAGGATCGAGTACTGGTCTACC

GGACTCTACAATATTGGGCCCTAAACCGTGAGTGACGCTGGCCATGCCTT

ACTTAGGTGGAGTTGGGCCGGTATCACTGACGGTATGAGTGCTCTCGGAC

TTTGGCTCGATTCAACCGACAGATGCTGCCCACGGCTGCCACGCCCTTCC

TCGCTCTGTTATCCCGTTCCTTTGTAACGGGATCGATTATATTGTGCTGT

ACCACGGAGTCTCGCTTGCATCGAGGGACGGCTTGTAGCGATAACACGCC

ATCAGGTGGTCCTCTCAGCTCTAACGCACGCTG

>ACGA_WT

GACGACCTTCACGACTCTCCTACATTCAGCACCTGCATCAGGGGCCAACG

ATACCATAGTGAGGGAACTAACGATTGCTCTTGGTGCACTTGAGTTCCCA

CGATGGCTGGCTGGACGAAGATATGGACCATTGATCACCAGGTGGTTAGA

GACCAAGACACTACTTAGTAAGGGAACTTTTTCATCTGATCTACCCTTGT

CTGGAAAAGAGAATTCACGAACGACACTGCCCAAGAAACTCTGCTAACGA

ATCATGCAGCCTCAATGTGGGATGACGAACGACCTTTTGCCACGACCTTA

CTGTTTGACATGCTGGGGTTTAACGAGACTGTCACGACTTGGAATGTTTC

CTCTTGAAGGTTAGCCCCCTGCTGTTAACGATTAGTTTCAATGGGAGTCT

ACACGAAACAGTGAGAAGGAACGACTTCATACCAAGAGCCCTTACAGTCT

CCCCTTTGGCACAGTCATACTGTCTTAGGAAGGCTTCATTGGCAGGTCAC

TATGGGATGTCCATCACGACTGCCTAAGAGCAGGCCACCTGACGATGAGT

GTGCCTTGAATTGACACGACCACGAAACGATTATCTGACAAACCCACGAT

GATGTACATGATAACGAAACGAACGAAGTTAGTACAACGATTCCTATCCA

GCAGGGAGGACCTTGCCTTGTTCACCTGACGAAAAAGGACGAAGAAAACG

ATAAGTCCAAACATAGGATCCTGAGTGGCTACTTCACGAACGAACAGGTC

AACGAACAGAGTCCAGTAAGACGAGACGATCAAGGCTATCACGATGCTTA

CTATTGATGGGGCAACACCTCTTCTACTACTTAATTTACGATGGTGATAG

GGATTGGCCAGACAGTTTGGATGCAGCATTGAGCTGGTTCCTTTACTTAT

GCTCTGAGGCCTACCACAGAGACGAGAATACACCATGATAAGGAGTTACC

TGTCTGTACCCTAACAGGCTGTAGCACGACTGATTGAAAGGGCATGGGAT

ACTTAGGCCCAGGATCAACGATAGGTCCCACGATATGGACTGGCACGATC

TTGCTTCACCCCCTGAAATGGGGGGGCTGAGAGGCCAAAGACGAACGATG

ACGATTTTCTAAGGCACACAATTTTACAATCTTGGATTATACAATCTGGT

ATCACGAGGTGCACTGATTGTGAGAGAACGACTGTTAACGATACAACGAC

CCCACTGGAGAATTGGCTGGCTAACGAACGATT

>CCGC_WT

GCCGCCATGGCCGCATCAGCTATTGAAAGAAACTGAGTTGGGCCAAGCCG

CTACAATAGTGAAATGAATACCGCTTGACATTGATGAGTTTGAGTCAACC

CGCCTGCTGGTTGGCCGCAGATAATGTCCAGGGACAAAGATGGAGTTAGC

TCTTCAGACAGTAGTTAGTAAGGCAAAGTTTTTCAACAAACTACAGTTTT

TTTGAAAAGAAAATGGCCGCCCGCCTGGGAAAAAGAAAGTCAGCTACCGC

TAAATGAAAACTGAAGGAGGCAGGCCGCCCGCAATTGATCCCCGCCAATA

TTGTCCCAAATGGTGGGCAATACCGCAGGGGAACCGCATGTGCAAATGAA

CAGACTGATGGTAGACACAAGCTGTTACCGCTTACTTTCTTTTCAAGTGT

AACCGCAAGAAGGAGCAGGACCGCAGCAATATGTTGCACTTGTAAGGTCT

GAATTTGGGCAAACTGATACAGTCATATGAATGGTTCATGAGCACTGCTT

TAGAGGATGTCCAATCCGCTTGACTAAGATCAGCATCTTGACCGCTGAAT

CAGCATTCAATTGTGCCGCCACCGCACCGCATATCCCACAAAACACCGCT

GTCATAAAGGTTACCGCCCCGCCCGCAACTAATAAGCCGCACACTAAATG

GATGGGAGGGAAGATGATTGTGATCCTGCCGCAATGAGCCGCAGAACCCG

CTATGAAGAAACTTAGTCAATTGAGAAACTATCATCCGCCCGCAGGGGAC

ACCGCACAAAGGCCCATAAACCGCTCCGCTTCTGGGTATCCCGCAACTTA

TTATCTTTGCTGCAACACATCTTGTACTACTTATTTTCCGCTGTGGATAG

GAGAATCCATTTCTGATTTGTTGAAGAAAAAAGACAATTTCTTTACTTAT

GGAGTGCAGCATAGGGAAGTGCCGCAGATAAAACCATGTAAGAAGATAAG

TGTGGGTACAATAAAAGCTTTTAGCCCGCCTGAAAAATTCAGCCTGGGTT

ACTTAGGCCCAGGGAGACCGCTAGATGGACCGCTATTGACTGGACCGCAT

TTGCTCCAGTTCATTTGAGATTGGGGACAAAGGGCCAAAGCCGCCCGCAT

CCGCTGTTTTAGGGCAAGCATTTTTATGATCTTGGAATATAATTTGAGGT

AATCCGCTGGAAGTTGATTGTGATTGACCGCTGAGTACCGCTAAGCCGCA

GGCACCAGACAGAAAGAATTCTACCGCCCGCTT

>GCGG_WT

GGCGGAATTGGCGGTTCAGGTATTTGAACAAACTCAATTTCTGGAATGCG

GTACAATACTGACCCAAATAGCGGCAGATCAGGGTGAGATTCAGAAAAGG

CGGTGCCTTGAGTGGCGGACATAATCTCCAGGGATGAAGATGAAGTTAGC

CATCAAGACTGTAGTTACTAAGACTCACATTTCCACTCAACTACAATTCT

TTTCAAAAACAAAGAGGCGGGCGGCTCCAAAAAAAAAAGTCAGCTAGCGG

TATTTGAACACTGAATGTGCTGCTGCGGGCGGGATCAACCAGCGGCATTA

TTCTCCCAAATCTTGATCAATAGCGGTCCCCCAGCGGATTTTCATGAGAA

CTTTCCCAGGTTACACACCTGCCATTAGCGGGTAAATTCAAGACTTCCTT

ATGCGGAAGACCCATCATGAGCGGAGTCATAGCCAGTTTTGGTACTCTTT

TGCCTTGGACAAATTCATACAGACTTAGGAAAGTTTCATCAGCCCTTCTC

TATTGGATGTCCAATGCGGCTGACTAACATCTGTCAAAACAGCGGTGAAT

TTGCATTCAAATCAGGCGGGAGCGGGGCGGATATCAAACATCAAAGCGGT

TCACTAAAGGTTAGCGGTGCGGGCGGGATTACTAAAGCGGACACTACAAA

CCAAGGTGAGAAGACCATTCTCAACATGGCGGAAAACAGCGGAGAAAGCG

GTAGGTGTCAGCATACCCTCCCCACTGACTAACCAGCGGGCGGCTTGCTC

AGCGGAAAAATCCACATACAGCGGAGCGGTTGTGGCTATGGCGGGACTTA

CTATGAACAGACCAACACATCATTTACTACATATCTTGCGGTGCAGATAC

TGGCAGACAATCATCTTTTCTGCAAGAAATGAGTGGGTGACTTTATGTAC

TCTGTCACTCCTACAAGAACAGCGGAAATAAAATCCCATATGAAAATAAC

TTTCTGTACAATAGAGCCCATTTAGGCGGCTCCACCATTCACCCTGCCAT

AAGTAGGCCCAGCCTCAGCGGTAGATCCAGCGGTATTCACCACTGCGGGT

TTCCTTCACCAAATTTCATTGGCCCCAACAAGCCACAACAGCGGGCGGAT

GCGGTTGTCTAAGATGTGCAAATTTAGAAACTTCCAATATAAGAAGAAAT

AAAGCGGTCTTCTCTGATTCTGATTCAGCGGCCCTTAGCGGTAGGCGGCA

GCAAAGAGACTGTTTCATCCTTAGCGGGCGGTT

>UCGU_WT

GTCGTAATTGTCGTTCAAGATAGGATGACACATGGAAGGGGGGGAGCTCG

TTACCATAAGGGCCCAAATATCGTCTGAACTGGGTTGGAGGCAGGGAACT

CGTTCCCAGCCTTGTCGTCAATAATGGACTTTGGTGAAGACCAAGTTAGC

CATCAAGACCATAGTTACTAAGAGAAACTGACAAACACAACTACAGTGCA

ATCAGAAAGAAAAGAGTCGTTCGTTGCTGCAAAATGAAGGGAGATATCGT

TCAAGAAACCCTCCATGGGTCACTTCGTTCGTCAGAGAACCTCGTCAGTA

TGCTCCCAAAGCCTGTGCAATATCGTGCCCCCATCGTGATCCCAGGGGAA

CTCTCCCAGGTTACACACTGGCCAGTATCGTGTAAGGTCAATGCAACTCT

AATCGTTGAACCCAGAAGGCTCGTGAGAATAAGGAGGAATGGTAAGCTCT

GGCAGGGGGAAAAGCCATACAGGGATAGGAAGGGTGCATCAGTCCTGCTG

TACAGGATGTGCAATTCGTCTGACTAACAGTGGGCTCAACATCGTTGAAG

AATTGGGGAAGAGAGTCGTCATCGTGTCGTCTAATGACTCAAACATCGTT

GGCCTAAGGGTTATCGTTTCGTTCGTGAGTACTAAATCGTGAACTAAATC

TCTCAAAGCAAAGAGCATGTGAAACCTGTCGTCACAACTCGTCAAGCTCG

TTAAAGATCAAGGTACTCACCCCACCAACTAATCTTCGTTCGTCTGGCCC

ATCGTCAAAACACTCATAACTCGTGTCGTTCAGCTCTATTTCGTGACATA

CTACAGACAGGCCAACACATGGGATACTACTTATGTTTCGTGGCCAGTAT

GGGCAGACAAGCTCAGTGGAGGGGAGAAACAAGAGTGTCACTTTACTTAC

ACATCCCCATCTACAAGACAATCGTGAATAAAACCCTTTATGATGATAAA

GGTCTGTACATTAGAGACATGTAGCTCGTCTGAACCAGGGATCCTGCCAT

ACCTAAGCCCAGCCTCATCGTTAGCCCAATCGTTAGGCAAGACATCGTGA

GCCCTTCACCAAAGTGCAAAGGCCTGACCAAGCCTCAAACTCGTTCGTCA

TCGTTGATTTATGAGAAGCAACTCTAAAAAGGGCCATTATAGGGAGACAT

AAGTCGTGGAGCCAGGACACAGGTTGATCGTCCCTTATCGTTAGCTCGTC

ACAAGCAGAGTGGGACACCCCTATCGTTCGTGG
