# Supplementary material for: Association of Zinc Finger Antiviral Protein Binding to Viral Genomic RNA with Attenuation of Replication of Echovirus 7
Source: mSphere. 2021 Jan 6;6(1):e01138-20. doi: 10.1128/mSphere.01138-20 (PMC7845596; doi:10.1128/mSphere.01138-20)
Supplement: TABLE S2 [file mSphere.01138-20_st002.docx]

TABLE S2

Primers used for E7 RNA quantitation

**Region ID ID Sequence (5'-3’)^1^ Effic.^2^ r^2^ Size**

E7 R1 CpG-H CpGH 345-549 F: AGCGATACGATGCAGACGC 95% 0.99 205

R: CGATGAACGGCGTGTCG

E7 R1 UpA-H UpAH 271-622 F: GCTGATGTACCCGCTACC 85% 0.998 352

F: ACCGTATCTGCTACCCTG

E7 R1 UpA-L R1 1054-1177 F: GGCAGACATCAACAAACCC  93% 0.98 124

R: GAAAAGTGTGACCACCCATC

E7 R1 WT R1 958-1122 F: GCACTAGCATAGCACAAGA  102% 0.994 165

+ CpG-L, CpG/UpA-L R: ATGGTATTGACATCCGTGG

5' UTR EQ-1 F: ACATGGTGTGAAGAGTCTATTGAGCT 96% 1 142

EQ-2 R: CCAAAGTAGTCGGTTCCGC

EP P: 6-FAM-TCCGGCCCCTGAATGCGGCTAAT-TAMRA

^1^Orientations - F: Forward; R: Reverse; P: Probe

^2^Amplification efficiency
